# Interdisciplinary problem-based learning model for standardized dental residency training: from theory to practice in dental trauma management

**DOI:** 10.3389/fmed.2024.1473943

**Published:** 2025-01-13

**Authors:** Yang Shuting, Wang Haohao, Wang Shida, Zheng Liwei, Wan Mian

**Affiliations:** ^1^State Key Laboratory of Oral Diseases and National Center for Stomatology and National Clinical Research Center for Oral Diseases, Department of Pediatric Dentistry, West China Hospital of Stomatology, Sichuan University, Chengdu, China; ^2^State Key Laboratory of Oral Diseases and National Center for Stomatology and National Clinical Research Center for Oral Diseases, Department of Cariology and Endodontic, West China Hospital of Stomatology, Sichuan University, Chengdu, China; ^3^State Key Laboratory of Oral Diseases and National Center for Stomatology and National Clinical Research Center for Oral Diseases, Department of General Practice, West China Hospital of Stomatology, Sichuan University, Chengdu, China

**Keywords:** interdisciplinary, standardized training of residents, problem-based learning model, dental trauma management, dentistry

## Abstract

**Objective:**

Enhancing clinical skills and quality of dental residents is critical for standardized training. Conventional standardized training falls short in exposing residents to diverse scenarios and fostering interdisciplinary collaboration, essential for dental trauma management. To address these issues, West China Hospital of Stomatology, Sichuan University introduced an interdisciplinary problem-based learning (IPBL) model to improve residents’ professional quality and practical abilities.

**Methods:**

Based on the specific needs of dental residents, the hospital established a PBL framework supported by an interdisciplinary collaborative teaching team with a focus on dental trauma diagnosis and treatment. A comprehensive assessment followed the IPBL course, which informed subsequent course modifications. In a preliminary study, 134 students voluntarily chose whether to participate in the program. At the end of the study, the scores of both groups were compared.

**Primary results:**

The IPBL model significantly improved the professional ability of dental residents in the diagnosis and treatment of dental trauma, and obtained positive evaluations from residents and mentors. For the quantitative analysis, the Wilcoxon signed-rank test showed an overall improvement in participants’ scores after completing the course.

**Conclusion:**

This teaching model provides a new way for the standardized training of dental residents, and helps to cultivate dental professionals with high professional and practical abilities.

## Introduction: background and rationale for the educational activity innovation

### Standardized dental residency training and the rationale for this educational innovation

Fostering general dentists capable of delivering exceptional dental services forms an important foundation of dental health needs in China (1). To address this, the National Health and Family Planning Commission of China, in collaboration with six other government departments, introduced the Standardized Residency Training (SRT) program in 2013. A dental student takes a 5-year undergraduate dental study and an additional 3-year SRT before practice independently. Seven dental specialties are included in the SRT program for dentistry. With this program, the dental residents are supposed to be equipped with strong professional ethics, communication skills, critical thinking abilities, self-motivation for continued education and training, and familiarity with dental practice regulations. The SRT program aims to help dental residents shift from theoretical education to hands-on clinical experience. However, conventional standardized training always fails to expose residents to a wide range of cases, making them feel unprepared and daunted when facing specific clinical scenarios. Dental trauma, in particular, poses a significant challenge for dental residents, due to the scarce opportunities to encounter such cases in dental school clinics or hospitals. Dental students and residents complain about the lack of uniformity and comprehensive coverage in dental trauma management training, leading to gaps in their practical skills and confidence when faced with real-world situations (2). Additionally, patients often express frustration with the variability in the quality of dental care they receive, particularly in managing complex dental trauma cases (1). To address these concerns and ensure high-quality dental care, it is crucial to improve the standardized training programs for dental residents.

### Challenges in training diagnosis and treatment of dental trauma

Most dental diseases necessitate interdisciplinary joint treatment, with dental trauma management being a typical example. Dental trauma management involves a range of diagnostic and treatment procedures, including history taking, clinical examination, pulp testing, radiographic examination, repositioning, splinting, endodontic treatment, coronal restoration, and aesthetic restoration. This approach integrates insights from various dental specialties including pediatric dentistry, oral and maxillofacial surgery, endodontics, periodontics, prosthodontics, orthodontics, and emergency oral medicine (3). Another consideration is the management of dental trauma among various specialists (4). Dental trauma cases, characterized by various dislocations, fractures, and specific pulp conditions, complicate the formulation of educational strategies. Mentoring residents in analyzing cases, diagnosing precisely, devising personalized treatment plans, and applying theory to practice remains essential. However, the current training system involves separate learning of distinct specialized contents. During the standardized training, residents rotate through various specialized departments separately. There is a noticeable absence of methods to interconnect these different specialized areas. Briefly, real-world clinical scenarios require interdisciplinary cooperation, but the present training system overlooks the development of interdisciplinary thinking and collaborative skills. Integrating multidisciplinary knowledge within the curriculum presents an educational challenge.

### Breaking down barriers: interdisciplinary education model

Given the multidisciplinary needs of diagnosis and treatment, an interdisciplinary educational model has been developed to provide residents with a thorough grasp of complex issues. This educational approach transcends traditional disciplinary boundaries, integrating knowledge across fields to deepen understanding. Interdisciplinary education in dentistry integrates courses from related disciplines into the dental curriculum (5). For instance, dental students take courses in medical sciences, anatomy, and physiology to gain a deeper understanding of the human body. Dental residents work alongside other specialties during clinical rotations, cooperating with specialists from different backgrounds. Dental residents are better equipped to provide comprehensive and patient-centered care through interdisciplinary study (6–10). The management of dental trauma often involves multiple specialties. The timely formulation of individualized treatment plans is crucial for the long-term prognosis of affected teeth. An interdisciplinary approach ensures the maintenance of both the aesthetic appeal and functional integrity of the rehabilitated area (11). However, it has been reported that dental trauma management is an area where residents have the least confidence in their abilities, and maxillofacial trauma is only superficially covered in the curriculum. A lack of knowledge in dental trauma management has also been noted among general dentists (2, 12–14). The interdisciplinary trauma curriculum has been highlighted as enhancing residents’ confidence in managing dental trauma. For standardized resident training, thus, a specific framework for an interdisciplinary curriculum in dental trauma management could be a crucial component of the standard training for dental residents, which needs to be clarified in dental trauma management training.

### What is driving the push for interdisciplinary problem-based learning in dental trauma management training?

Problem-Based Learning (PBL) is an instructional approach that emphasizes active learning through the exploration and resolution of real-world problems. It originated in the 1960s at McMaster University in Canada, pioneered by Dr. Howard Barrows, and has since gained widespread adoption in medical and dental education worldwide (15–18). Active learning yields superior educational outcomes, offering social–emotional support, reducing student anxiety, and fostering a deeper understanding alongside academic benefits (19). The advantages of PBL include residents’ critical thinking, problem-solving skills, self-directed learning abilities, and teamwork competencies. These advantages make PBL an ideal implementation form of the interdisciplinary model. Interdisciplinary problem-based learning (IPBL) is an innovative educational approach that integrates knowledge and techniques from multiple disciplines to solve complex, real-world problems (20–22). In the context of dental trauma management training, IPBL offers several unique advantages. Firstly, IPBL significantly enhances residents’ critical thinking and problem-solving skills. Under the expert guidance of experienced tutors, residents collaborate in small groups to analyze cases in-depth, identify key issues, and formulate hypotheses. They then conduct comprehensive research using literature reviews, expert consensus, and clinical guidelines to develop a new knowledge system and practical solutions. By analyzing complex dental trauma cases and synthesizing information from various sources—including patient history, clinical examinations, pulp testing, and radiographic examinations—residents hone their critical thinking abilities and make well-informed decisions about diagnosis and treatment plans (23, 24). Secondly, IPBL promotes interdisciplinary collaboration among residents. Dental trauma management often requires input from multiple specialties, such as dental emergency care, pediatric dentistry, oral and maxillofacial surgery, endodontics, and prosthodontics. By bringing together tutors and residents from diverse backgrounds to work on shared cases, IPBL fosters a more comprehensive understanding of trauma management and encourages effective interdisciplinary collaboration (25, 26). Lastly, IPBL prepares residents for the demands of clinical practice. This approach not only helps residents develop strong clinical skills but also equips them with transferable skills such as interdisciplinary clinical thinking, active learning, evidence-based decision-making, cross-disciplinary collaboration, and a commitment to lifelong learning (27). By staying current with the latest technological advancements, these well-rounded dentists can provide timely, evidence-based care, ultimately leading to improved patient outcomes. In conclusion, the IPBL framework represents a cutting-edge educational strategy that can help dental residents develop both clinical skills and essential transferable skills. We propose IPBL as an effective means of enhancing dental residents’ clinical competence and ethical standards (28).

## Pedagogical frameworks, pedagogical principles, standards underlying the educational activity

### Establishing IPBL model

The teaching team initially establishes the IPBL teaching model, outlining curriculum objectives, delivery methods, group structure, and grading systems. Combining lectures, case studies, and practical exercises, the curriculum enables dental residents to master various aspects of dental care including trauma diagnosis, emergency and endodontic treatments, restorative plans, and prognoses. It also focuses on fostering evidence-based and interdisciplinary clinical reasoning by discussing controversial issues, analyzing treatment methods, and evaluating new treatments. Additionally, the curriculum seeks to develop interdisciplinary collaboration skills, urging residents to partner with various dental specialists. This collaborative approach aids residents in formulating comprehensive plans for patients and improving communication with other dental professionals. Residents are encouraged to participate in research, attend seminars and workshops on dental trauma and cultivate a lifelong learning attitude.

In the SRT dentistry program at West China Hospital of Stomatology, Sichuan University, a focused IPBL curriculum on dental trauma management was conducted. Under the classic grouping model, each group included 6–10 first-year dental residents from general dentistry and endodontics, led by an experienced mentor. Acting as facilitators, mentors guide residents through curriculum materials, facilitate discussions, and provide performance feedback. Residents needed to demonstrate mastery of the curriculum objectives and their ability to apply this knowledge in practical settings. Grades were determined through a comprehensive evaluation process, including self, peer, and mentor assessments. These include: adherence to discipline, rules, and regulations; a positive attitude; the quality and quantity of clinical work, along with efficiency in task completion; operating skills; patient communication skills (no patient complaints); cooperation and teamwork in the department; ability to communicate and coordinate with the outside world; resilience; self-learning; and clinical discourse skills. This thorough evaluation system guarantees a fair assessment of residents’ performance (29).

### The “script” for IPBL curriculum: curriculum plans and case studies

**Figure 1:**
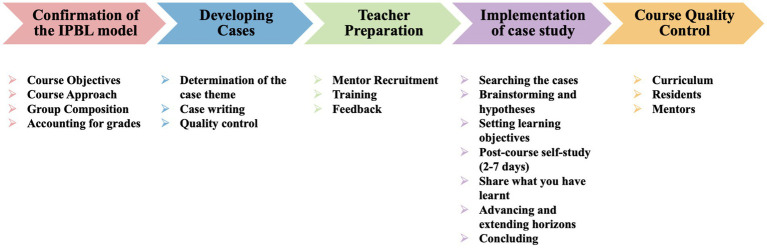
Framework of the interdisciplinary problem-based learning (IPBL) curriculum.

The teaching plan, acting as a ‘script’ for IPBL curricula, aims to boost residents’ learning enthusiasm (see Figure 1). It details the curriculum’s flow, steering residents through various learning activities. The experienced teaching team carefully selected dental trauma case for its educational value. They compiled cases based on the typical clinical scenarios, ensuring that residents were exposed to a broad spectrum of situations they may encounter in their practice. To facilitate effective learning, the teaching team strategically provides case information in stages throughout the curriculum. This progressive approach allows residents to build their knowledge and understanding gradually, simulating the process of clinical decision-making. The case information includes detailed descriptions of the patient’s disease progression, current medical history, past history, and pertinent examination results. This comprehensive information enables residents to develop a holistic understanding of the case and practice their diagnostic and treatment skills in a realistic and context-rich environment.

Taking the curriculum “Diagnosis and Treatment of Young Permanent Teeth Fracture” as an example (see Supplementary material 1), experienced educators and clinicians from General Dentistry, the Department of Pediatric Dentistry, and the Department of Cariology and Endodontics compiled a case based on a young permanent tooth fracture in an 8-year-old female. This case covered problems related to the diagnosis of tooth fracture, endodontic treatment of young permanent teeth, strategies for coronal restoration, post-treatment guidelines, determination of follow-up intervals, and prevention of young permanent teeth trauma. It was designed to enhance the learning experience and develop a deeper understanding of young permanent teeth fracture management. By providing a structured framework and engaging case-based learning opportunities, it aimed to empower residents with the knowledge and skills necessary to excel in their clinical practice and provide optimal care to their patients with dental trauma.

## Learning environment; learning objectives; pedagogical format

### The IPBL mentoring team

An effective mentoring team need develop a multifaceted learning environment for dental residents and ultimately achieves the established teaching goals. We recruited mentors from various specialties, including general practice, dental emergency, pediatric dentistry, endodontics, orthodontics, prothodontics, implant dentistry and oral and maxillofacial Surgery. For one of the courses, “Diagnosis and Treatment of Young Permanent Teeth Fracture,” the mentoring team was mainly composed of clinicians from general practice, pediatric dentistry, and endodontics. Mentors from general practice introduced clinical examination and radiographic examination. Mentors from pediatric dentistry were responsible for the characteristics of young permanent teeth and endodontic strategies. Endodontists covered the diagnosis of tooth fracture and the coronal restoration strategies post-endodontic treatment. Mentors from dental emergency focused on the emergency management of post-traumatic dental injuries. Severe tooth dislocation or cases with minimal retention significance may require subsequent orthodontic, restorative, implant, or surgical interventions.

Training programs were conducted to equip our mentors with the necessary skills and knowledge to effectively guide and support the dental residents throughout the curriculum. This training focused on:

1. Ensuring the orderly progression of IPBL and encouraging critical thinking.
2. Promoting active student engagement in all aspects of the curriculum and ensuring that all residents have an opportunity to contribute and learn from each other.
3. Facilitating effective discussions among residents and achieving the established goals in the teaching plan.

To continuously improve our mentoring team, we evaluated the success of the mentoring by tracking student progress, assessing their knowledge and skills through exams and practical assessments, monitoring their engagement and satisfaction with the curriculum, rewarding outstanding mentors who excelled in the curriculum, and using the outcomes and evaluations to inform future iterations and improvements of the mentoring program (30).

### The implementation and execution of the case study

**Figure 2:**
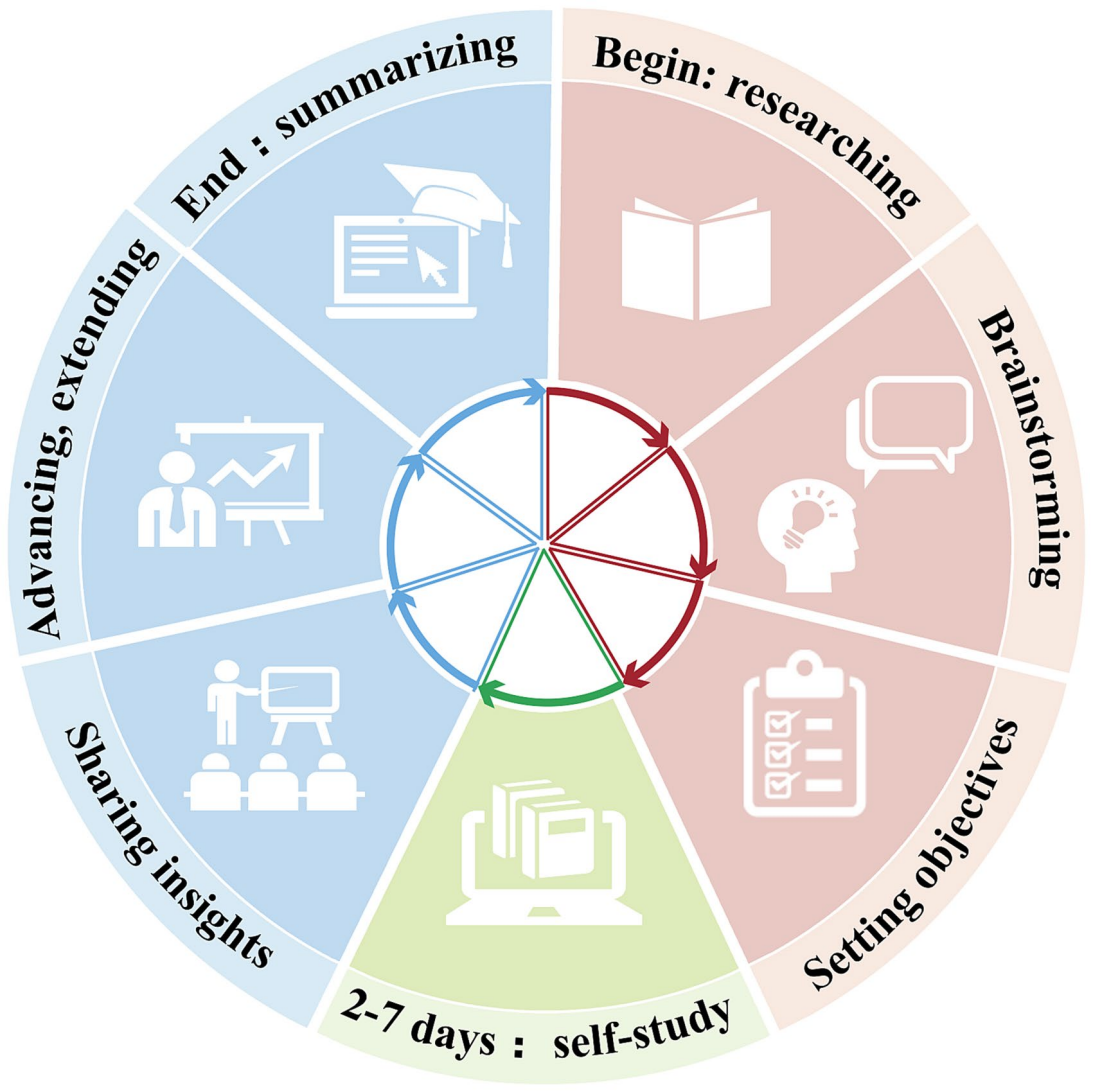
The IPBL case study implementation is executed in three phases. Phase 1: getting to know each other, establishing rules, researching cases; brainstorming and hypothesizing; setting learning objectives. Phase 2: 2–7 days of post-course learning. Phase 3: sharing what has been learnt; advancing and extending horizons; finally, summarizing knowledge.

The case study is typically conducted over a two-day period, totaling 4 academic hours of structured classroom time (see Figure 2). The first day is dedicated to introductory content, where residents are provided with a thorough overview of the case. The case itself is designed to be rich in information, including the patient’s medical history, the specific context surrounding the dental trauma, the results of preliminary examinations such as x-rays and physical assessments, and routine imaging studies performed to aid in diagnosis. Based on the information provided, residents are prompted to raise questions actively. They are assigned learning objectives that aim to deepen their understanding in dental trauma management. These key objectives include diagnosing dental trauma, deciding on endodontic treatments, and determining timing and strategies for coronal restoration. The first day’s class sets a solid foundation, enabling residents to explore these objectives through discussions and activities. Following the first day’s class, residents receive 2–7 days for self-study, focusing on the objectives, reviewing case materials, and conducting further research. This period of self-study enhances residents’ knowledge, applies theoretical concepts practically, and prepares them for the next case study phase.

**Figure 3:**
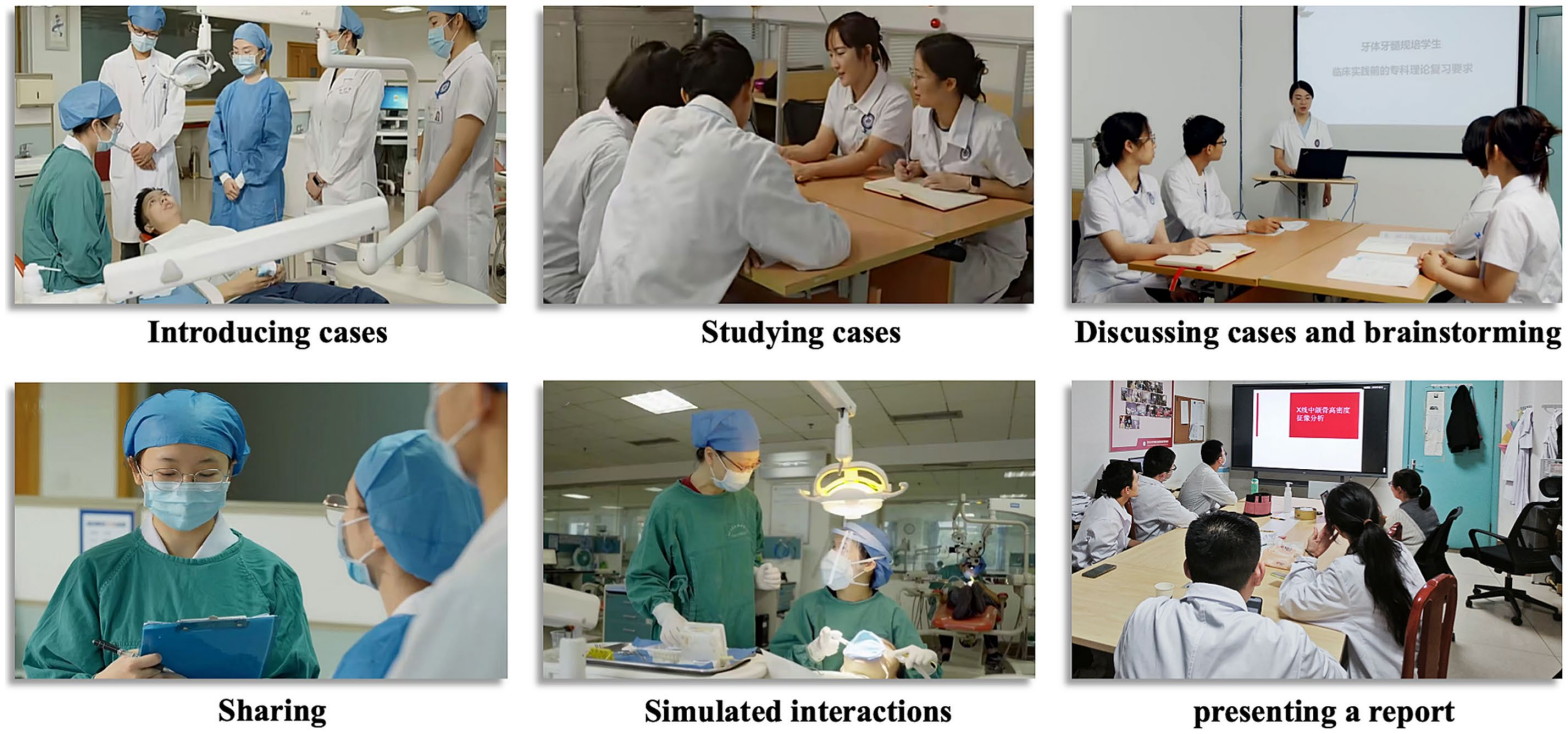
Illustrations of the implementation process.

On the second day of the curriculum, the focus shifts to the presentation of the residents’ self-study results. They were required to present their understanding of the case and demonstrate their learning progress. Presentations included interpreting dental trauma, engaging in discussions, applying theory to practical scenarios through simulated patient interactions, and presenting dental trauma case reports. Using new case materials provided, residents are challenged to provide accurate diagnoses, formulate comprehensive final treatment plans, communicate effectively with patients about invasive procedures, and ultimately conclude the case study by presenting their findings, recommendations, and rationale for their decisions (see Figure 3).

### Evaluation and feedback

After the successful implementation of the IPBL curriculum in dental trauma learning, the teaching team embarked on a comprehensive evaluation process. This multi-faceted approach encompassed diverse methods aimed at providing a thorough understanding of the effectiveness and impact of the IPBL model (see Supplementary materials 2, 3). Firstly, residents were assessed through various metrics, including their participation in group discussions, critical thinking abilities demonstrated during case analyses, and their ability to integrate knowledge from different dental specialties. This assessment helped identify areas of fields requiring further development. Mentors were evaluated based on their teaching approach and guidance in critical thinking and interdisciplinary collaboration. These evaluations aimed to ensure that mentors were equipped with the competency to lead the IPBL. A specific mentor-student evaluation system was established as well. Residents give suggestions according to their learning experiences, such as what worked well and what should be improved. Mentors provided feedback on residents’ performance and addressed areas for further growth. These allowed for the continuous improvement of the curriculum to align with the needs of the residents.

With the feedback gathered from these evaluations, the teaching team performed an analysis of the IPBL curriculum. Targeted adjustments were made to both the cases and curriculum. The case studies were updated to ensure they covered a broader range of dental trauma cases, including hard dental tissue and pulp injury, periodontal injury, and supporting bone injury. This diversity aimed to prepare residents for the challenges they would encounter in their future clinic work. The curriculum was modified to enhance the learning objectives according to the feedback. The results were also adapted to improve the mentor-guided discussions in promoting critical thinking and problem-solving skills.

### Preliminary results to date

In a preliminary study, 268 students voluntarily chose whether to participate in the program. The questionnaires for both the experimental group and the teacher were administered and collected through SoJump.com (wjx.cn; in short: SoJump), and the scores from both groups were subsequently compared (see Supplementary materials 4–6). All statistical analyses were performed with SPSS 26.0, and the level of significance was set at *p* < 0.05. Notably, the results were statistically significant, with the Wilcoxon rank-sum test and independent sample t-tests showing an overall improvement in participants’ scores after completing the course (Table 1; Supplementary material 7). In the first stage, students chose whether to participate in the IPBL project based on their preferences and interests. To ensure equal learning opportunities, IPBL courses were also offered to students who initially opted out. The mean age of the control group was 24.62 ± 1.35 years, while the average age of the experimental group was 24.51 ± 1.38 years. The experimental group consisted of 66% females and 34% males, whereas the control group comprised 65% females and 35% males.

**Table 1:** Comparison of scores of students who participated in the IPBL program and those who did not.

	Scores	Z	*p*
Experimental groups	86 (78, 92)	−5.213	0.000
Control groups	92 (86, 96)		

Upon conducting the IPBL curriculum of dental trauma management, both mentors and residents offered positive feedback. Overall, the course received high ratings on the Likert scale (1–5), with most categories scoring above 4 and a general appreciation rating of 4.2 (Table 2). We acknowledge that the interpretation of the table is descriptive; however, evaluating both educational and general aspects is critical to assess the success of a new course. When introducing a new curriculum with innovative, active teaching and learning methods, it is vital to assess their efficacy and ensure their meaningful impact. Residents highlighted the integration of theoretical knowledge from diverse disciplines. This approach significantly enhanced their confidence when confronted with dental trauma cases in clinic. They further suggested that expanding the curriculum to include additional cases and content related to periodontology and oral and maxillofacial surgery would further enrich their learning experience. Most residents reported that IPBL significantly enhanced their understanding of the complexity and multidisciplinary nature of dental trauma management, improved their critical thinking and problem-solving abilities, and rated the experience highly. A resident from pediatric dentistry remarked, “The case study allowed me to experience a near-real scenario of dental trauma. I was particularly impressed by the case of the ‘complicated crown-root fracture of anterior teeth,’ which required a combination of oral surgery and endodontics due to palatal trauma fracture and gap loss. A joint consultation with orthodontics and prosthodontics was needed to develop a comprehensive plan.” A resident from department of cariology and endodontics suggested, “This course deepened our understanding of trauma, providing a clearer impression of treatment steps. I was previously worried about handling these types of traumas in the future independent practice, but now I feel more confident dealing with traumatic dental injuries.” Another resident from general dentistry appreciated the curriculum, stating, “There is no greater feeling than experiencing it firsthand; it is very different from textbook knowledge, since real-life cases can be very complicated. They require flexibility, tolerance, and understanding from the patient’s perspective.”

**Table 2:** Survey questionnaire on the interdisciplinary problem-based learning model in dental trauma learning.

Questions	1–5 Likert (1 = strongly disagree, 5 = strongly agree)					Average *N* = 84
	1	2	3	4	5	
Understand comprehensively	0	3	8	38	35	4.25
Facilitate your learning	2	3	23	23	33	3.98
Learning goals are clear	1	4	14	32	33	4.10
Cases are comprehensive	2	8	21	18	35	3.90
Collaboration is excellent	2	3	26	21	32	3.93
There is an improvement in the ability to link theory to practice	1	5	9	37	32	4.12
Critical thinking and problem-solving skills have improved	1	2	10	34	37	4.24
Be confident in managing dental trauma cases independently	1	2	15	41	25	4.04
Be satisfied with PBL	0	3	11	36	34	4.20

The mentoring team echoed these views, noting substantial improvements in residents’ diagnostic and treatment skills, reflected in an overall rating of 4.13 (Table 3). Questions related to perceptions of the Interdisciplinary PBL Model also received high scores. This means a significant increase in the ability of residents to handle complex dental trauma cases with a more comprehensive and interdisciplinary perspective, and the success of IPBL in forming critical thinking, problem-solving, and interdisciplinary collaboration. Most mentors believed that the teaching cases “covered the complexity of dental trauma cases” and “interaction with the residents inspired new thoughts and insights.” For instance, mentors from the department of pediatric dentistry found “it is interesting to analyze the clinical and epidemiological characteristics of dental trauma in children with the residents.” They observed that “the occurrence of dental trauma in children has regularity in terms of gender, age, and seasons, and children often do not seek timely medical consultation for dental trauma. Strengthening health education on dental trauma in children is essential to reduce the probability of dental injuries.” An orthodontist noted that “the optimal method and timing of early fixed orthodontic treatment for traumatized teeth are still controversial, “prompting debates with residents on “the effects of tooth movement, direction of orthodontic force application, and type of orthodontic appliance on traumatized teeth.” These discussions also laid the foundation for the residents’ future clinical work.

**Table 3:** Teacher’s survey on the interdisciplinary problem-based learning model in dental trauma learning.

Questions	1–5 Likert (1 = strongly disagree, 5 = strongly agree)					Average *N* = 32
	1	2	3	4	5	
PBL enhances residents’ understanding	0	3	5	11	13	4.06
The integration of multiple disciplines is effective	1	2	4	14	11	4.00
PBL helps foster interdisciplinary collaboration	1	3	3	9	16	4.13
The quality of the cases is excellent	1	3	8	12	8	3.72
Cases are comprehensive and complex	1	4	6	11	10	3.78
The instructor-guided discussions promote critical thinking and problem-solving skills	2	2	2	11	15	4.09
Provide feedback on residents’ performance timely	0	1	12	13	6	3.75
Be satisfied with PBL	0	5	4	12	11	3.91

## Discussion on the practical implications, objectives and lessons learned

To address the limitations of traditional training methods, particularly in the area of dental trauma management, where residents often struggled due to inadequate exposure to diverse case scenarios and limited interdisciplinary collaboration, West China Hospital of Stomatology, Sichuan University introduced the IPBL model into the standardized training of dental residents. Our results indicated that the IPBL model significantly improved residents’ proficiency in dental trauma management, particularly in the diagnosis and treatment of dental injuries. This outcome is attributed to IPBL. IPBL residents were actively engaged in problem-solving. It promoted critical thinking and problem-solving skills, as evidenced by their ability to analyze cases, make accurate diagnoses, and formulate individualized treatment plans. The interdisciplinary model bridges gaps in knowledge among various dental specialties. Dental trauma management includes fields such as dental emergency, pediatric dentistry, oral and maxillofacial surgery, endodontics, periodontics, prosthodontics, and orthodontics. Integrating specialists from these disciplines, the IPBL facilitates a comprehensive understanding of dental trauma management for residents. Regular interdisciplinary workshops and training sessions can form a cohesive learning environment within the SRT program.

The evaluation process was instrumental in refining the IPBL curriculum. Using a comprehensive evaluation system that included residents, mentors, and the curriculum itself, we identified improvement areas and made necessary adjustments. Positive feedback from residents and mentors affirms the curriculum’s effectiveness. Residents reported growing confidence in managing dental trauma, while mentors observed notable progress in residents. Despite these encouraging outcomes, further improvements are still required. Residents proposed the inclusion of additional disciplines like periodontics and oral surgery for a broader perspective. Broadening case studies to include more scenarios would enhance residents’ clinical experience. The evaluation maintains the curriculum up-to-date in dental medicine and addresses emerging educational needs. Additionally, employing technology and digital tools can improve the PBL experience. For example, incorporating online platforms and virtual simulations can provide residents with access to a wider range of dental trauma cases and facilitate remote collaboration among team members. This approach not only enriches their learning experience but also makes the program more flexible and accessible. Virtual-based clinical cases are more efficient and popular with students than in-person tutorials (31). We are also developing a virtual interactive clinical platform app that assigns roles such as “doctor” and “patient.” This app uses animations to recreate the entire process of trauma treatment, including “consultation and history taking, oral examination and result analysis, diagnosis, treatment design, preoperative communication, treatment process, postoperative advice, prognosis, and regular follow-up.” Each stage aims to realistically reproduce clinical scenes and refine the steps involved. For instance, in a class on “pulpotomy and crown bonding, “specific operations like “local anesthesia, isolation of the operative area, pulp opening, pulpotomy, pulp capping, tooth preparation, and resin aesthetic restoration” are shown sequentially. Residents carry out the entire process themselves, selecting the appropriate instruments and focusing on key treatment points. This approach uses vivid and engaging clinical case studies to strengthen residents’ theoretical knowledge and develop their clinical and independent thinking skills.

Dental training and medical student education have also evolved in recent years. The flipped classroom model has been effective in enhancing pediatric dental students’ theoretical understanding (32). Debating has similarly improved students’ communication and reasoning abilities (33). Advances in digital technology and artificial intelligence offer novel platforms for student development, such as digital 3D technologies (34), while also highlighting the need for critical and effective utilization of these innovations (35, 36).

Lastly, emphasizing lifelong learning and self-directed study is also crucial in dental SRT program. IPBL emphasizes the active role of residents in the study group and the whole SRT program. By engaging in self-directed study, attending conferences, and participating in professional development programs, residents can continue to expand their knowledge and skills beyond the IPBL curriculum.

Team-based learning (TBL) has gained traction in medical education and may represent an advancement over PBL (37). Centered on small student groups, TBL enhances interaction and communication, fostering student initiative and promoting sustainable learning (38–40). Despite its potential for transformative impact, challenges such as teacher preparation, resource allocation, and effective assessment remain. Continuous refinement of learning methods and technologies is essential to adapt to the evolving healthcare environment (41).

No educational process marks the end, marking the onset of ongoing learning. Future steps for the IPBL curriculum in the Dental SRT program at West China Hospital of Stomatology include continuous evaluation and refinement, curriculum expansion, enhanced interdisciplinary collaboration, and increased technology integration. These initiatives guarantee the IPBL’s sustained efficacy within the Dental SRT program.

### Acknowledgment of any conceptual, methodological, environmental, or material constraints

There are some limitations to this paper. For example, no Randomized Controlled Trial (RCT) methodology was used, which makes it difficult to say whether the training program actually provided tangible benefits. The sample size is relatively small. A larger number of participants, strictly adhering to RCT methodology, are needed. Timely feedback collection and an extended follow-up period would also help to strengthen the evidence presented.

## Conclusion

The IPBL is crucial in dental trauma education, proficiently preparing dental residents with the requisite knowledge, skills, and attitudes for managing dental trauma. It promotes collaboration, integration, and critical thinking and offers a novel approach to standardized training for dental residents, nurturing professionals with advanced capabilities.

## Data Availability

The original contributions presented in the study are included in the article/Supplementary material, further inquiries can be directed to the corresponding author.
