# Supplementary material for: Interdisciplinary problem-based learning model for standardized dental residency training: from theory to practice in dental trauma management
Source: Front Med (Lausanne). 2025 Jan 13;11:1473943. doi: 10.3389/fmed.2024.1473943 (PMC11770602; doi:10.3389/fmed.2024.1473943)
Supplement: Supplementary file 1 [file Table_1.docx]

**Supplementary material 1**

**Diagnosis and Treatment of Young Permanent Teeth Fracture**

The patient with Trauma to the Left Upper Front Tooth

**I. Case Background**

The patient presented to the pediatric dental clinic accompanied by her parents due to "trauma to the left upper front tooth sustained for approximately 24 hours". The patient is otherwise healthy, with no reported history of systemic diseases, allergies, or previous dental trauma. The incident occurred during a fall while playing outdoors.

**II. Problem Description**

The patient reported falling on her face, resulting in immediate pain and sensitivity in the left upper front tooth region. Upon examination, the following observations were made:


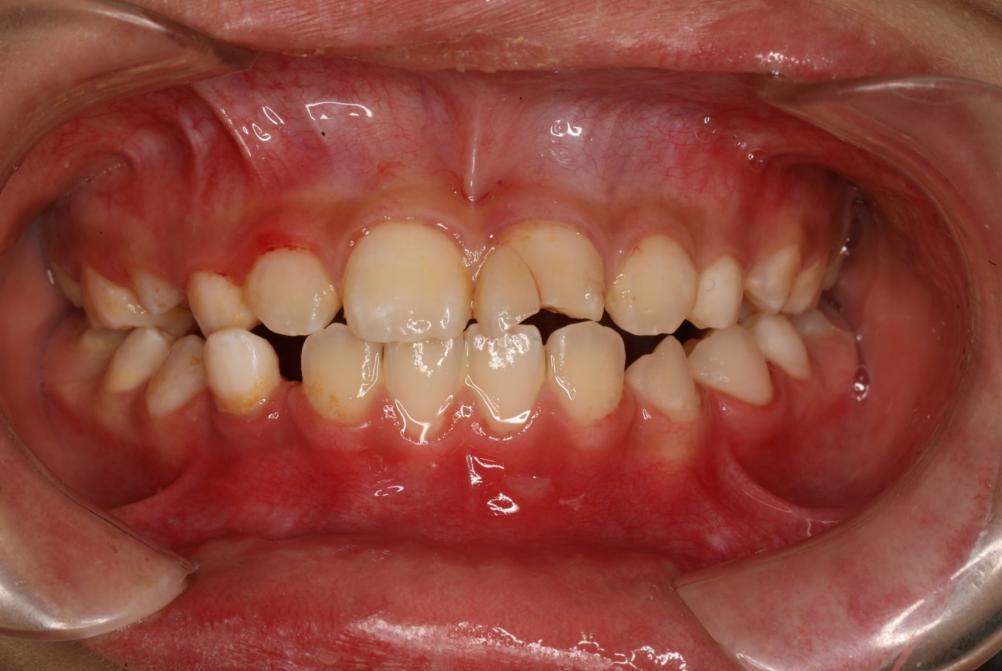

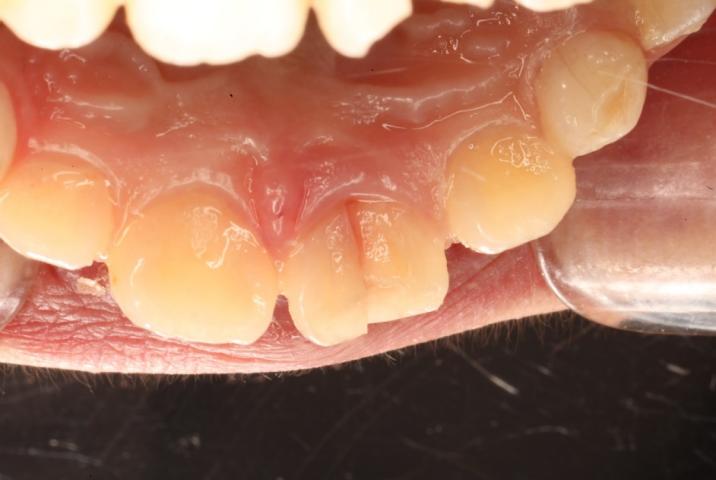


（Provided by Dr. Li Yiting from West China Hospital of Stomatology）

**Extraoral Examination:** No facial asymmetry or swelling noted.

**Intraoral Examination:**

No alveolar bone fracture detected through palpation.

Soft tissues appeared intact without any signs of laceration or bruising.

Tooth #21 (left upper central incisor) exhibited a fracture line involving both enamel and dentin, with notable defects at the mesial and distal incisal edges.

A longitudinal crack was visible in the crown, extending below the gingival margin, exposing the pulp cavity.

The tooth responded normally to a cold test, indicating pulp vitality.

Percussion test elicited a positive/negative response, suggesting possible periodontal involvement.

Gingival bleeding was observed around the affected tooth.

The mesial fragment of the fractured tooth displayed Grade II mobility.

**Imaging Examination:**

**
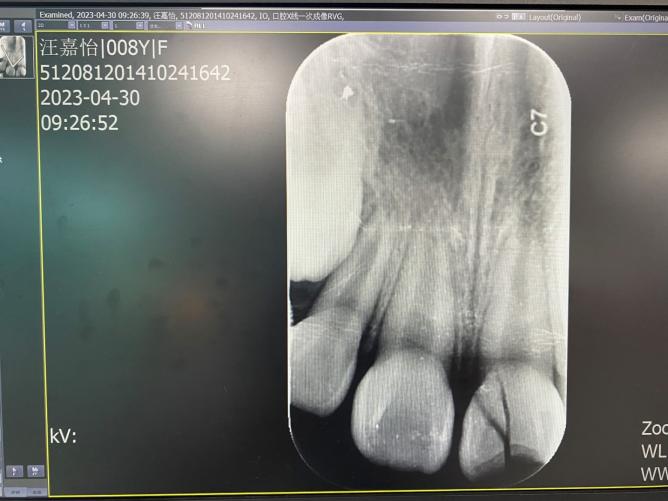
**

（Provided by Dr. Li Yiting from West China Hospital of Stomatology）

Radiographic images revealed two longitudinal fracture lines within the crown of tooth #21, extending down to the cervical one-third of the root.

Root length was nearly fully developed, with a wide-open apical foramen indicating incomplete apical closure.

No periapical radiolucency was detected, ruling out apical periodontitis.

There were no signs of root fracture or alveolar bone fracture.

**III. Problem Formulation**

Given the patient's clinical symptoms and radiographic findings, the following questions may arise:

1. Diagnosis of Tooth #21

How to accurately diagnose Tooth #21?

1. Selection of Endodontic Treatment

When confronted with the need for endodontic treatment, how can we make an informed decision? Should the priority be on preserving pulp vitality, or is root canal treatment necessary?

1. Strategies for Coronal Restoration

What are the effective methods for performing coronal restoration to ensure the restoration of tooth function and appearance?

1. Post-treatment Guidelines

What specific instructions and recommendations should be provided to patients following treatment?

1. Determining Follow-up Intervals

How can we appropriately determine the follow-up intervals to ensure timely monitoring of the patient's recovery progress?

1. Prevention of Young Permanent teeth trauma

What strategies and preventive measures can be implemented to reduce the incidence of similar dental traumas, especially for young permanent teeth among child patients?

**IV. Learning Objectives**

The primary objectives of this PBL case are:

To gain proficiency in the classification and diagnostic methodologies of dental trauma.

To learn and master the fundamental principles and techniques for endodontic treatment of traumatic young permanent teeth.

To understand and evaluate various factors that influence endodontic treatment planning, ensuring the continued development of the roots of young permanent teeth.

To learn and master the strategies and techniques for coronal restoration of traumatic young permanent teeth.

To explore and comprehend strategies for preventing dental trauma in children, focusing on education, awareness, and environmental modifications.

**V. Learning Steps**

Group Discussion: Each student assigned to discuss and provide answers to the formulated questions.

Literature Review: Each student will conduct a thorough review of relevant scientific literature to support their diagnostic and treatment plan recommendations.

Treatment Plan Formulation: Based on their discussions and literature review, each group will develop a detailed treatment plan tailored to the patient's needs.

Presentation and Discussion: Each group will present their proposed treatment plan to the class, followed by a facilitated discussion to exchange ideas and perspectives.

Teacher's Comments: The instructor will offer constructive feedback on each group's treatment plan, highlighting strengths and suggesting areas for improvement.

**VI. Evaluation and Feedback**

Group Evaluation: Each group will self-evaluate their collaboration, communication skills, and ability to work effectively as a team during the discussion and treatment planning process.

Teacher's Evaluation: The instructor will assess students' comprehension of dental trauma concepts, their critical thinking skills in formulating treatment plans, and their ability to innovate within the realm of dental trauma management.
