# Supplementary material for: Interdisciplinary problem-based learning model for standardized dental residency training: from theory to practice in dental trauma management
Source: Front Med (Lausanne). 2025 Jan 13;11:1473943. doi: 10.3389/fmed.2024.1473943 (PMC11770602; doi:10.3389/fmed.2024.1473943)
Supplement: Supplementary file 2 [file Table_2.docx]

**Supplementary material 2**

**Survey Questionnaire on the Interdisciplinary Problem-Based Learning Model in Dental Trauma Learning**

Dear Participants,

We are conducting this survey to gather your feedback on the interdisciplinary problem-based learning (PBL) model implemented in our dental trauma learning program. Your responses will be invaluable in helping us improve and refine this educational approach. Please take a few minutes to answer the following questions honestly and thoroughly.

**Section 1: General Information**

1. What is your current year of residency training?
   1. First Year
   2. Second Year
   3. Third Year or Above
2. What specialty are you pursuing within dental medicine?
   1. General Dentistry
   2. Oral and Maxillofacial Surgery
   3. Pediatric Dentistry
   4. Endodontics
   5. Prosthodontics
   6. Periodontics
   7. Orthodontics
   8. Other (please specify): ________________________

**Section 2: Interdisciplinary PBL Experience**

1. How satisfied were you with the interdisciplinary approach taken in the PBL sessions for dental trauma learning?
   1. Very Satisfied
   2. Satisfied
   3. Neutral
   4. Dissatisfied
   5. Very Dissatisfied
2. Did the interdisciplinary PBL model help you understand the complexity and multidisciplinary nature of dental trauma management?
   1. Yes, significantly
   2. Somewhat
   3. Neutral
   4. Not much
   5. No
3. How effective was the interdisciplinary team in facilitating your learning?
   1. Very Effective
   2. Effective
   3. Neutral
   4. Ineffective
   5. Very Ineffective

**Section 3: Course Structure and Content**

1. How clear were the course objectives and learning goals related to dental trauma management?
   1. Very Clear
   2. Clear
   3. Neutral
   4. Unclear
   5. Very Unclear
2. Did the PBL cases cover a diverse range of dental trauma scenarios, including repositioning, splinting, endodontic treatment, coronal restoration, and later aesthetic restoration?
   1. Yes, comprehensively
   2. Yes, to some extent
   3. Neutral
   4. No, not enough
   5. No, not at all
3. How did you find the group composition and collaboration within your PBL team?
   1. Excellent
   2. Good
   3. Neutral
   4. Poor
   5. Very Poor

**Section 4: Learning Outcomes**

1. To what extent did the interdisciplinary PBL model enhance your ability to integrate knowledge from different dental specialties?
   1. Greatly Enhanced
   2. Somewhat Enhanced
   3. Neutral
   4. Not Much Enhanced
   5. Not Enhanced at All
2. Did the PBL sessions improve your critical thinking and problem-solving skills in dental trauma management?
   1. Yes, significantly
   2. Yes, somewhat
   3. Neutral
   4. No, not much
   5. No, not at all
3. How confident do you feel in managing dental trauma cases independently after completing the PBL program?
   1. Very Confident
   2. Confident
   3. Neutral
   4. Not Very Confident
   5. Not Confident at All

**Section 5: Feedback and Suggestions**

1. What were the most valuable aspects of the interdisciplinary PBL model for your learning?
2. What areas, if any, do you think could be improved in the interdisciplinary PBL program for dental trauma learning?
3. Do you have any suggestions for incorporating additional disciplines or topics into the PBL sessions to further enhance your learning experience?

**Conclusion**

Thank you for taking the time to complete this survey. Your feedback is crucial in helping us optimize the interdisciplinary PBL model for dental trauma learning. We appreciate your honest responses and will use them to continually improve our educational programs.
