# Supplementary material for: Interdisciplinary problem-based learning model for standardized dental residency training: from theory to practice in dental trauma management
Source: Front Med (Lausanne). 2025 Jan 13;11:1473943. doi: 10.3389/fmed.2024.1473943 (PMC11770602; doi:10.3389/fmed.2024.1473943)
Supplement: Supplementary file 3 [file Table_3.docx]

**Supplementary material 3**

**Teacher's Survey on the Interdisciplinary Problem-Based Learning Model in Dental Trauma Learning**

Dear Teacher,

We are conducting a survey to gather your insights and feedback on the interdisciplinary problem-based learning (PBL) model implemented in dental trauma learning at our institution. Your responses will be invaluable in helping us evaluate the effectiveness of this teaching approach and identify areas for further improvement. Please take a few minutes to complete the following questionnaire.

**Section 1: General Information**

1. **Department/Specialty**:
2. **Years of Experience in Dental Education**:

________ years

**Section 2: Perception of the Interdisciplinary PBL Model**

1. **To what extent do you agree that the interdisciplinary PBL model enhances dental residents' understanding of dental trauma?**
   1. Strongly Agree
   2. Agree
   3. Neutral
   4. Disagree
   5. Strongly Disagree
2. **How effective do you find the integration of multiple disciplines (e.g., oral emergency, pediatric dentistry, oral and maxillofacial surgery) in the PBL sessions?**
   1. Very Effective
   2. Somewhat Effective
   3. Neutral
   4. Not Very Effective
   5. Ineffective
3. **Does the PBL model help foster interdisciplinary collaboration among residents and instructors?**
   1. Yes, significantly
   2. Yes, to some extent
   3. Neutral
   4. No, not much
   5. No, not at all

**Section 3: Teaching and Learning Experience**

1. **How would you rate the quality of the case studies used in the PBL sessions?**
   1. Excellent
   2. Good
   3. Average
   4. Poor
   5. Very Poor
2. **Do you think the case studies adequately cover the range and complexity of dental trauma cases?**
   1. Yes, fully
   2. Yes, partially
   3. Neutral
   4. No, not much
   5. No, not at all
3. **How effective are the instructor-guided discussions in promoting critical thinking and problem-solving skills among residents?**
   1. Very Effective
   2. Somewhat Effective
   3. Neutral
   4. Not Very Effective
   5. Ineffective

**Section 4: Evaluation and Feedback**

1. **How often do you provide feedback to residents on their performance in PBL sessions?**
   1. Always
   2. Often
   3. Sometimes
   4. Rarely
   5. Never
2. **What are the primary challenges you face in implementing the interdisciplinary PBL model?** (Open-ended)
3. **What suggestions do you have for improving the interdisciplinary PBL model in dental trauma learning?** (Open-ended)

**Section 5: Overall Satisfaction**

1. **Overall, how satisfied are you with the interdisciplinary PBL model for dental trauma learning?**
   1. Very Satisfied
   2. Satisfied
   3. Neutral
   4. Dissatisfied
   5. Very Dissatisfied

**Thank you for taking the time to complete this survey. Your input is crucial to our continuous improvement efforts.**
