# Supplementary material for: Interdisciplinary problem-based learning model for standardized dental residency training: from theory to practice in dental trauma management
Source: Front Med (Lausanne). 2025 Jan 13;11:1473943. doi: 10.3389/fmed.2024.1473943 (PMC11770602; doi:10.3389/fmed.2024.1473943)
Supplement: Supplementary file 4 [file Table_4.docx]

**Supplementary material 4. Some of the Course feedback from residents**

2023.10-2024.1

| Specialty | Remarks |
| --- | --- |
| Pediatric dentistry | The case study allowed me to experience a near-real scenario of dental trauma. I was particularly impressed by the case of the 'complicated crown-root fracture of anterior teeth,' which required a combination of oral surgery and endodontics due to palatal trauma fracture and gap loss. A joint consultation with orthodontics and prosthodontics was needed to develop a comprehensive plan. |
| Dental emergency | I learnt methods and techniques of doctor-patient communication and developed a sense of professional ethics and responsibility |
| Endodontics | I have actively learnt the relevant theoretical knowledge and put it into practice in the clinic. However, my communication skills with patients need to be further improved, my proficiency in clinical operation needs to be acquired through exercise, and my awareness of protection during operation needs to be improved. |

2024.1-2024.4

| Specialty | Remarks |
| --- | --- |
| Endodontics | This course deepened our understanding of trauma, providing a clearer impression of treatment steps. I was previously worried about handling these types of traumas in the future independent practice, but now I feel more confident dealing with traumatic dental injuries. |
| Oral and maxillofacial Surgery | I have made some progress in diagnosis. Through study and practice, I have been able to more accurately identify the signs and symptoms of complex trauma, improving the accuracy and reliability of diagnosis. |
| Orthodontics | I have developed my coping skills, improved my clinical thinking and gained clinical experience from various teaching activities. |

2024.4-2024.7

| Specialty | Remarks |
| --- | --- |
| General dentistry | There is no greater feeling than experiencing it firsthand; it is very different from textbook knowledge, since real-life cases can be very complicated. They require flexibility, tolerance, and understanding from the patient's perspective. |
| Prothodontics | Through step-by-step analyses of clinical features and identification of diseases, PBL encourages in-depth thinking and discussion, giving me, as a prosthodontics student, a deeper understanding of trauma. |
| Implant dentistry | I have acquired many clinical skills, and in addition to hands-on practice, I have learnt many communication skills and become more responsible and empathetic. |
