# Supplementary material for: Interdisciplinary problem-based learning model for standardized dental residency training: from theory to practice in dental trauma management
Source: Front Med (Lausanne). 2025 Jan 13;11:1473943. doi: 10.3389/fmed.2024.1473943 (PMC11770602; doi:10.3389/fmed.2024.1473943)
Supplement: Supplementary file 5 [file Table_5.docx]

**Supplementary material 5. Course feedback from residents**

|  | Q1 | Q2 | Q3 | Q4 | Q5 | Q6 | Q7 | Q8 | Q9 | Q10 | Q11 | Q12 | Q13 | Q14 |
| --- | --- | --- | --- | --- | --- | --- | --- | --- | --- | --- | --- | --- | --- | --- |
| 1 | C.第三年 | D.牙体牙髓病科 | B.满意 | B.是，有一定程度 | A.非常有效 | B.清晰 | A.是，全面覆盖 | A.优秀 | A.很大提升 | A.是的，非常显著 | A.非常自信 | 这门课程加深了我们对创伤的理解，使我们对治疗步骤有了更清晰的印象。我之前曾担心在未来的独立实践中处理此类创伤的问题，但现在我对处理牙科创伤更有信心了。（This course deepened our understanding of trauma, providing a clearer impression of treatment steps. I was previously worried about handling these types of traumas in the future independent practice, but now I feel more confident dealing with traumatic dental injuries.） | Limited funding to support the development of high-quality, interdisciplinary case materials. | Create smaller, more focused groups to improve the quality of discussions and participation. |
| 2 | A.第一年 | G.正畸科 | A.非常满意 | B.是，有一定程度 | A.非常有效 | B.清晰 | A.是，全面覆盖 | A.优秀 | A.很大提升 | B.是的，有一点 | A.非常自信 | 我提高了应对能力，改进了临床思维，并通过各种教学活动积累了临床经验。（I have developed my coping skills, improved my clinical thinking and gained clinical experience from various teaching activities.） | Difficulty in evaluating the effectiveness of the interdisciplinary PBL model objectively. | Incorporate emerging technologies, like virtual reality, to simulate complex trauma cases. |
| 3 | B.第二年 | E.修复科 | C.中立 | C.中立 | D.无效 | D.不清晰 | D.否，不够多 | D.不太好 | D.提升不多 | C.中立 | C.中立 | 在辩论问题上很有启发 | no | no |
| 4 | C.第三年 | F.牙周病学 | C.中立 | C.中立 | C.中立 | C.中立 | D.否，不够多 | C.中立 | D.提升不多 | C.中立 | C.中立 | 通过思维碰撞获得新的发现 | no | no |
| 5 | C.第三年 | G.正畸科 | C.中立 | C.中立 | C.中立 | C.中立 | D.否，不够多 | C.中立 | C.中立 | C.中立 | C.中立 | 自我学习有助于思考 | no | no |
| 6 | A.第一年 | E.修复科 | A.非常满意 | A.是的，非常显著 | A.非常有效 | B.清晰 | A.是，全面覆盖 | B.好 | A.很大提升 | A.是的，非常显著 | A.非常自信 | 通过对临床特征和疾病的逐步分析和识别，PBL鼓励深入思考和讨论，作为一名修复学学生，这让我对创伤有了更深刻的理解。（Through step-by-step analyses of clinical features and identification of diseases, PBL encourages in-depth thinking and discussion, giving me, as a prosthodontics student, a deeper understanding of trauma.） | Insufficient collaboration between faculty members from different departments. | Foster a culture of open communication and mutual respect among interdisciplinary teams. |
| 7 | B.第二年 | A.全科 | C.中立 | C.中立 | C.中立 | C.中立 | D.否，不够多 | C.中立 | C.中立 | C.中立 | C.中立 | 阅读大量的病例辅助了我的学习 | no | no |
| 8 | A.第一年 | A.全科 | C.中立 | C.中立 | C.中立 | C.中立 | D.否，不够多 | C.中立 | C.中立 | C.中立 | C.中立 | 通过讨论和总结对我的学习最有帮助 | no | no |
| 9 | C.第三年 | A.全科 | B.满意 | B.是，有一定程度 | C.中立 | C.中立 | C.中立 | C.中立 | B.是，有一定程度提升 | B.是的，有一点 | C.中立 | 通过发言、与他人交流培养了我的思维能力 | no | no |
| 10 | A.第一年 | G.正畸科 | B.满意 | B.是，有一定程度 | C.中立 | C.中立 | C.中立 | C.中立 | B.是，有一定程度提升 | B.是的，有一点 | C.中立 | 通过大量的案例学习让我更加得心应手 | no | no |
| 11 | A.第一年 | C.儿童口腔医学 | A.非常满意 | A.是的，非常显著 | A.非常有效 | A.非常清晰 | A.是，全面覆盖 | B.好 | B.是，有一定程度提升 | A.是的，非常显著 | B.自信 | 案例研究让我体验了接近真实场景的牙外伤。我对“前牙复杂冠根折”的案例印象尤为深刻，该案例因腭侧创伤骨折和间隙丢失需要结合口腔外科和牙髓病学进行治疗。此外，还需要正畸学和修复学的联合会诊来制定综合治疗计划。（The case study allowed me to experience a near-real scenario of dental trauma. I was particularly impressed by the case of the 'complicated crown-root fracture of anterior teeth,' which required a combination of oral surgery and endodontics due to palatal trauma fracture and gap loss. A joint consultation with orthodontics and prosthodontics was needed to develop a comprehensive plan.） | no | no |
| 12 | B.第二年 | B.口腔颌面外科 | D.不满意 | D.不太多 | D.无效 | D.不清晰 | D.否，不够多 | C.中立 | D.提升不多 | D.否，不太多 | D.不太自信 | 与来自不同学科的同伴合作的机会拓宽了我的视野，并提高了解决问题的能力。 | no | no |
| 13 | A.第一年 | C.儿童口腔医学 | B.满意 | B.是，有一定程度 | C.中立 | C.中立 | C.中立 | C.中立 | B.是，有一定程度提升 | B.是的，有一点 | C.中立 | 最有价值的方面是融合了多样化的视角，促进了协作解决问题的能力，并将理论知识应用于实际情境中。 | The program could benefit from more hands-on clinical scenarios and a clearer structure to balance theoretical content with practical experience. | Incorporating topics like psychology or trauma counseling could help enhance the understanding of patient care and improve decision-making in trauma situations. |
| 14 | C.第三年 | F.牙周病学 | B.满意 | B.是，有一定程度 | C.中立 | C.中立 | C.中立 | C.中立 | B.是，有一定程度提升 | B.是的，有一点 | B.自信 | 与来自不同学科的同伴合作的机会拓宽了我的视野，并提高了解决问题的能力。 | There could be more emphasis on hands-on clinical practice to complement the theoretical learning. | Incorporating more on medical ethics would help in decision-making during complex trauma cases. |
| 15 | A.第一年 | C.儿童口腔医学 | B.满意 | B.是，有一定程度 | C.中立 | B.清晰 | C.中立 | C.中立 | B.是，有一定程度提升 | B.是的，有一点 | B.自信 | 真实的案例研究让学习更加有趣和相关，弥合了理论与实践之间的差距。 | The feedback process could be more structured to help students improve and reflect on their progress. | Introducing public health topics related to trauma prevention would help contextualize dental care within the broader healthcare system. |
| 16 | B.第二年 | F.牙周病学 | B.满意 | B.是，有一定程度 | C.中立 | B.清晰 | C.中立 | C.中立 | B.是，有一定程度提升 | B.是的，有一点 | B.自信 | 与来自不同领域的学生一起工作让我学会了如何从多个角度处理问题。 | The case studies could incorporate more diverse scenarios, including rare or complex trauma cases. | A focus on legal aspects, such as liability in trauma cases, could help students understand the full scope of their responsibilities. |
| 17 | A.第一年 | B.口腔颌面外科 | B.满意 | B.是，有一定程度 | C.中立 | B.清晰 | C.中立 | C.中立 | B.是，有一定程度提升 | B.是的，有一点 | B.自信 | 这增强了团队合作和沟通能力，而这些是任何专业领域的关键技能。 | More time could be dedicated to individual reflection and understanding the learning process. | Including surgical techniques relevant to dental trauma would enrich the practical aspects of learning. |
| 18 | A.第一年 | D.牙体牙髓病科 | B.满意 | B.是，有一定程度 | C.中立 | B.清晰 | C.中立 | C.中立 | B.是，有一定程度提升 | B.是的，有一点 | B.自信 | 这种模式通过整合各种学术学科，提供了对牙外伤更全面的认识。 | The integration of more advanced technology in simulations could further enhance learning. | Including more about advanced diagnostic tools and imaging technologies would help broaden the approach to trauma care. |
| 19 | C.第三年 | E.修复科 | C.中立 | B.是，有一定程度 | C.中立 | C.中立 | C.中立 | C.中立 | C.中立 | C.中立 | C.中立 | 在处理案例的思路上更加清晰。 | no | no |
| 20 | B.第二年 | H.其他：急诊 | A.非常满意 | B.是，有一定程度 | A.非常有效 | A.非常清晰 | A.是，全面覆盖 | A.优秀 | A.很大提升 | A.是的，非常显著 | A.非常自信 | 学习了医患沟通的方法和技巧，并培养了专业伦理和责任感。（I learnt methods and techniques of doctor-patient communication and developed a sense of professional ethics and responsibility） | collaboration | no |
| 21 | A.第一年 | D.牙体牙髓病科 | C.中立 | B.是，有一定程度 | C.中立 | C.中立 | C.中立 | C.中立 | C.中立 | B.是的，有一点 | C.中立 | 在总结一个完整的牙外伤病例中我学到了很多 | no | no |
| 22 | A.第一年 | F.牙周病学 | B.满意 | B.是，有一定程度 | C.中立 | C.中立 | D.否，不够多 | C.中立 | C.中立 | B.是的，有一点 | C.中立 | 我通过测试加强了知识点的巩固和积累 | no | no |
| 23 | A.第一年 | F.牙周病学 | B.满意 | B.是，有一定程度 | C.中立 | B.清晰 | C.中立 | C.中立 | B.是，有一定程度提升 | B.是的，有一点 | B.自信 | 跨学科的方法培养了批判性思维，并使我们能够创造性地解决问题。 | Some sessions could benefit from having clearer learning objectives to ensure focus and clarity. | Incorporating elements of nutrition and its impact on recovery could provide a more holistic approach to trauma treatment. |
| 24 | C.第三年 | E.修复科 | B.满意 | B.是，有一定程度 | C.中立 | B.清晰 | C.中立 | C.中立 | B.是，有一定程度提升 | B.是的，有一点 | B.自信 | 学习如何整合医学、心理学和牙科学知识帮助我从多角度理解创伤护理。 | The pace of the program could be adjusted to allow more in-depth exploration of complex cases. | Introducing topics related to the use of robotics in dental care could bring innovative technology into the learning environment. |
| 25 | A.第一年 | D.牙体牙髓病科 | B.满意 | B.是，有一定程度 | B.有效 | B.清晰 | C.中立 | C.中立 | B.是，有一定程度提升 | B.是的，有一点 | B.自信 | 接触来自不同领域专业人士的观点让我变得更加适应性强且思想开放。 | More opportunities for hands-on practice, particularly in dental techniques, would be beneficial. | It would be beneficial to include topics on cross-cultural differences in trauma care, especially for international settings. |
| 26 | A.第一年 | C.儿童口腔医学 | B.满意 | B.是，有一定程度 | B.有效 | B.清晰 | C.中立 | C.中立 | B.是，有一定程度提升 | B.是的，有一点 | B.自信 | 在专家指导下将理论知识应用于实际情境提供了宝贵的机会。 | It would be helpful to have more structured opportunities for peer feedback and collaboration. | The integration of more medical imaging technologies could improve diagnostic and treatment strategies for trauma cases. |
| 27 | B.第二年 | D.牙体牙髓病科 | B.满意 | B.是，有一定程度 | B.有效 | B.清晰 | C.中立 | C.中立 | B.是，有一定程度提升 | B.是的，有一点 | B.自信 | 我获得了在压力下工作和管理跨学科项目的实践技能。 | The inclusion of real-world guest speakers or professionals in dental trauma could bring practical insights into the sessions. | Offering more on systemic health conditions that affect trauma recovery would be valuable in treating patients holistically. |
| 28 | A.第一年 | D.牙体牙髓病科 | B.满意 | B.是，有一定程度 | C.中立 | B.清晰 | C.中立 | C.中立 | B.是，有一定程度提升 | B.是的，有一点 | B.自信 | 我积极学习了相关的理论知识并将其应用于临床实践。然而，我在患者沟通能力方面仍需进一步提升，临床操作熟练度需要通过练习来获得，同时在操作过程中对防护的意识也需要加强。（I have actively learnt the relevant theoretical knowledge and put it into practice in the clinic. However, my communication skills with patients need to be further improved, my proficiency in clinical operation needs to be acquired through exercise, and my awareness of protection during operation needs to be improved.） | A more balanced integration of academic and practical content would enhance the overall learning experience. | Introducing topics on data analysis and machine learning in medical decision-making could prepare students for future technological advances. |
| 29 | A.第一年 | C.儿童口腔医学 | A.非常满意 | A.是的，非常显著 | B.有效 | A.非常清晰 | A.是，全面覆盖 | A.优秀 | A.很大提升 | B.是的，有一点 | A.非常自信 | 我发现将实践和理论学习结合在实时场景中的方式特别有益。 | The amount of reading material could be reduced to avoid information overload. | Including topics on advanced pain management and anesthetic techniques would be helpful for dealing with traumatic dental injuries. |
| 30 | C.第三年 | G.正畸科 | A.非常满意 | A.是的，非常显著 | B.有效 | A.非常清晰 | B. 是，有一定程度覆盖 | A.优秀 | A.很大提升 | A.是的，非常显著 | A.非常自信 | 该项目帮助我培养了批判性思维能力，并在团队环境中有效沟通。 | More interdisciplinary workshops or seminars would help reinforce the collaborative nature of trauma care. | A focus on dental trauma management in pediatric patients would provide important knowledge for working with younger populations. |
| 31 | A.第一年 | G.正畸科 | B.满意 | B.是，有一定程度 | C.中立 | B.清晰 | C.中立 | C.中立 | B.是，有一定程度提升 | B.是的，有一点 | B.自信 | 学术知识与实际应用的结合让我获得了未来实践所需的技能。 | The integration of psychological aspects of trauma could be improved by including more related content. | A more in-depth focus on pharmacology and pain management in dental trauma could enhance understanding of treatment options. |
| 32 | C.第三年 | A.全科 | B.满意 | B.是，有一定程度 | C.中立 | B.清晰 | C.中立 | C.中立 | B.是，有一定程度提升 | B.是的，有一点 | B.自信 | 以团队为基础的方法帮助我发展了领导能力和协作能力。 | There should be a clearer breakdown of each discipline’s role within the PBL process. | Topics on patient communication and managing trauma-induced anxiety could improve how students interact with patients. |
| 33 | A.第一年 | E.修复科 | A.非常满意 | A.是的，非常显著 | B.有效 | A.非常清晰 | B. 是，有一定程度覆盖 | A.优秀 | A.很大提升 | A.是的，非常显著 | A.非常自信 | 跨学科的环境让我在牙外伤治疗方案上获得了多样化的视角。 | Additional support in case analysis and reflection could further improve students' critical thinking. | Adding topics on disaster management and mass casualty care could prepare students for high-pressure trauma scenarios. |
| 34 | A.第一年 | D.牙体牙髓病科 | A.非常满意 | A.是的，非常显著 | B.有效 | A.非常清晰 | B. 是，有一定程度覆盖 | A.优秀 | A.很大提升 | B.是的，有一点 | A.非常自信 | 与来自不同领域的专家讨论病例的机会是这一学习模式的重要收获之一。 | More structured case debriefs could enhance learning outcomes and help clarify complex concepts. | Introducing topics related to trauma counseling and psychological support would enhance patient-centered care. |
| 35 | B.第二年 | C.儿童口腔医学 | A.非常满意 | B.是，有一定程度 | A.非常有效 | A.非常清晰 | B. 是，有一定程度覆盖 | A.优秀 | A.很大提升 | B.是的，有一点 | A.非常自信 | PBL让我看到了我的研究领域与其他学科及更广泛医疗系统的关联性。 | Sessions could include more case-based discussions that reflect common real-world challenges. | Including topics on geriatric trauma care could prepare students for working with older populations. |
| 36 | A.第一年 | H.其他：种植科 | A.非常满意 | A.是的，非常显著 | B.有效 | A.非常清晰 | A.是，全面覆盖 | B.好 | A.很大提升 | B.是的，有一点 | A.非常自信 | 我掌握了许多临床技能，除了实践操作外，我还学习了许多沟通技巧，并变得更加负责任和富有同理心。（I have acquired many clinical skills, and in addition to hands-on practice, I have learnt many communication skills and become more responsible and empathetic.） | Maintaining student engagement and motivation throughout complex, interdisciplinary cases. | Provide case follow-ups to demonstrate the long-term impact of decisions made during PBL sessions. |
| 37 | A.第一年 | F.牙周病学 | A.非常满意 | A.是的，非常显著 | B.有效 | A.非常清晰 | A.是，全面覆盖 | B.好 | A.很大提升 | B.是的，有一点 | A.非常自信 | 主动学习模式鼓励了自主研究，加深了我对牙外伤的理解。 | Incorporating more role-playing exercises could help build empathy and practical skills. | More coverage of advanced imaging techniques, like 3D scanning for dental injuries, could enhance diagnostic skills. |
| 38 | A.第一年 | A.全科 | A.非常满意 | B.是，有一定程度 | A.非常有效 | A.非常清晰 | A.是，全面覆盖 | A.优秀 | B.是，有一定程度提升 | A.是的，非常显著 | A.非常自信 | 我通过处理实际案例获得了实践经验，提升了我的实践知识。 | Some students might benefit from having more targeted learning resources based on their specific areas of interest. | Incorporating topics on legal documentation and how to handle trauma cases from a legal standpoint could help students navigate real-world cases. |
| 39 | A.第一年 | D.牙体牙髓病科 | A.非常满意 | A.是的，非常显著 | B.有效 | A.非常清晰 | A.是，全面覆盖 | B.好 | A.很大提升 | B.是的，有一点 | A.非常自信 | 该项目的协作性质帮助我优化了团队合作能力。 | It would be helpful to integrate a broader variety of trauma cases beyond just dental trauma. | Including nutrition and diet management in trauma recovery could provide a more holistic approach to patient care. |
| 40 | A.第一年 | C.儿童口腔医学 | A.非常满意 | A.是的，非常显著 | B.有效 | A.非常清晰 | A.是，全面覆盖 | A.优秀 | B.是，有一定程度提升 | B.是的，有一点 | A.非常自信 | 基于案例的学习让我在实时情境中发展了解决问题的能力。 | There should be more focus on trauma prevention strategies alongside treatment approaches. | The integration of emergency response and trauma triage systems would improve students' preparedness for real-world scenarios. |
| 41 | B.第二年 | D.牙体牙髓病科 | B.满意 | A.是的，非常显著 | A.非常有效 | A.非常清晰 | B. 是，有一定程度覆盖 | A.优秀 | A.很大提升 | A.是的，非常显著 | B.自信 | 与来自不同学科的他人一起学习让我对创伤护理有了更全面的理解。 | The model could include more opportunities for personalized learning paths based on individual strengths and weaknesses. | Topics on patient advocacy and social work within the trauma field could help students understand their role in patient recovery. |
| 42 | A.第一年 | B.口腔颌面外科 | A.非常满意 | A.是的，非常显著 | A.非常有效 | B.清晰 | B. 是，有一定程度覆盖 | A.优秀 | A.很大提升 | B.是的，有一点 | A.非常自信 | 通过团队合作和集体解决问题，我增强了处理复杂案例的信心。 | More interaction with dental trauma specialists could help provide deeper insights into the field. | More focus on the management of facial and maxillofacial trauma cases could broaden the knowledge base. |
| 43 | A.第一年 | C.儿童口腔医学 | A.非常满意 | B.是，有一定程度 | A.非常有效 | A.非常清晰 | B. 是，有一定程度覆盖 | A.优秀 | A.很大提升 | B.是的，有一点 | A.非常自信 | 跨学科PBL模式让我深入了解了创伤护理的各个方面，而不仅限于牙科治疗。 | The use of virtual simulations could be expanded to provide more interactive learning opportunities. | It could be beneficial to introduce topics related to patient insurance and billing in trauma care. |
| 44 | A.第一年 | F.牙周病学 | A.非常满意 | B.是，有一定程度 | A.非常有效 | A.非常清晰 | B. 是，有一定程度覆盖 | A.优秀 | A.很大提升 | B.是的，有一点 | A.非常自信 | 该项目鼓励了批判性思维和在治疗策略方面的创新。 | It would be beneficial to have clearer timelines and milestones for completing tasks and assignments. | Including sessions on advanced wound care and infection control in trauma care would enhance practical learning. |
| 45 | A.第一年 | G.正畸科 | A.非常满意 | B.是，有一定程度 | A.非常有效 | A.非常清晰 | B. 是，有一定程度覆盖 | A.优秀 | A.很大提升 | B.是的，有一点 | A.非常自信 | 与不同背景的专业人士合作让我学习了多样化的诊断和治疗方法。 | The group sizes could be adjusted to make collaboration more efficient and productive. | Providing exposure to multidisciplinary trauma response teams could help students understand the collaborative nature of trauma care. |
| 46 | A.第一年 | C.儿童口腔医学 | A.非常满意 | A.是的，非常显著 | B.有效 | A.非常清晰 | A.是，全面覆盖 | B.好 | A.很大提升 | B.是的，有一点 | A.非常自信 | PBL模式提供了更个性化的学习，兼顾了个人和团队的需求。 | The program could incorporate more community-based learning to understand trauma care in different environments. | A focus on integrative medicine and complementary therapies in trauma recovery could provide students with additional approaches to care. |
| 47 | A.第一年 | A.全科 | D.不满意 | D.不太多 | E.非常无效 | E.Very Unclear | E. 否，完全没有覆盖 | E.非常不好 | E. 完全没有提升 | E. 否，完全没有覆盖 | E.根本不自信l | 通过与他人讨论完善了自己的认识 | no | no |
| 48 | B.第二年 | B.口腔颌面外科 | D.不满意 | D.不太多 | E.非常无效 | D.不清晰 | E. 否，完全没有覆盖 | E.非常不好 | D.提升不多 | D.否，不太多 | D.不太自信 | 案例的解读与体验让我对牙外伤的体会更加深入 | no | no |
| 49 | A.第一年 | A.全科 | A.非常满意 | B.是，有一定程度 | A.非常有效 | B.清晰 | A.是，全面覆盖 | A.优秀 | A.很大提升 | A.是的，非常显著 | B.自信 | 没有什么比亲身体验更令人感受深刻；这与教科书知识非常不同，因为现实中的案例可能非常复杂。它们需要灵活性、宽容以及从患者角度出发的理解。（There is no greater feeling than experiencing it firsthand; it is very different from textbook knowledge, since real-life cases can be very complicated. They require flexibility, tolerance, and understanding from the patient's perspective.） | Time constraints in preparing and coordinating interdisciplinary PBL sessions. | Establish a mentorship program where students can learn from professionals in different fields. |
| 50 | A.第一年 | D.牙体牙髓病科 | C.中立 | C.中立 | D.无效 | D.不清晰 | D.否，不够多 | D.不太好 | D.提升不多 | C.中立 | C.中立 | 与他人讨论让我受益 | no | no |
| 51 | A.第一年 | B.口腔颌面外科 | C.中立 | C.中立 | C.中立 | C.中立 | C.中立 | D.不太好 | C.中立 | C.中立 | C.中立 | 与同学辩论打开了我的思路 | no | no |
| 52 | A.第一年 | F.牙周病学 | A.非常满意 | B.是，有一定程度 | A.非常有效 | A.非常清晰 | A.是，全面覆盖 | B.好 | A.很大提升 | A.是的，非常显著 | A.非常自信 | 我很感激跨学科模式让我为医疗工作中协作的本质做好了准备。 | The feedback loop could be faster to allow for more timely adjustments in learning. | Introducing global health perspectives on trauma care would broaden students' understanding of healthcare systems in different contexts. |
| 53 | B.第二年 | D.牙体牙髓病科 | A.非常满意 | B.是，有一定程度 | A.非常有效 | A.非常清晰 | B. 是，有一定程度覆盖 | B.好 | B.是，有一定程度提升 | A.是的，非常显著 | B.自信 | 这一经历帮助我理解了理论、实践和现实案例之间的相互联系。 | Incorporating more real-time data analysis could make the learning experience more relevant. | Including mental health and PTSD management in trauma recovery would improve the psychological aspects of care. |
| 54 | A.第一年 | G.正畸科 | A.非常满意 | A.是的，非常显著 | A.非常有效 | A.非常清晰 | A.是，全面覆盖 | B.好 | A.很大提升 | B.是的，有一点 | B.自信 | 该项目强调了协作的重要性，帮助我学会了如何与他人高效合作。 | Providing more case follow-ups after the sessions would help students see the long-term outcomes of their decisions. | Offering more interdisciplinary interactions with medical professionals, like emergency responders, would enrich learning. |
| 55 | B.第二年 | D.牙体牙髓病科 | A.非常满意 | B.是，有一定程度 | B.有效 | A.非常清晰 | A.是，全面覆盖 | A.优秀 | A.很大提升 | B.是的，有一点 | A.非常自信 | PBL让我能够参与更深入的讨论，并从多个角度学习创伤护理。 | More case discussions focusing on non-trauma-related aspects of dental care could offer a more balanced approach. | The inclusion of bioethics in trauma care could provide a moral framework for decision-making. |
| 56 | C.第三年 | A.全科 | A.非常满意 | B.是，有一定程度 | B.有效 | A.非常清晰 | B. 是，有一定程度覆盖 | B.好 | B.是，有一定程度提升 | B.是的，有一点 | A.非常自信 | 学术理论与临床实践的整合让我能够立即将所学应用于实际。 | The program could include more role-specific training within interdisciplinary teams to clarify each professional's role. | Topics related to trauma care in rural or underserved settings could give students a more diverse view of healthcare delivery. |
| 57 | B.第二年 | D.牙体牙髓病科 | A.非常满意 | B.是，有一定程度 | B.有效 | A.非常清晰 | B. 是，有一定程度覆盖 | B.好 | A.很大提升 | A.是的，非常显著 | A.非常自信 | 跨学科方法帮助我更好地理解了各专业人士在创伤护理中如何协同工作。 | Incorporating more case-sharing among institutions could provide different perspectives on treatment approaches. | The integration of trauma care in sports-related injuries could enhance the understanding of specific trauma management. |
| 58 | B.第二年 | C.儿童口腔医学 | A.非常满意 | B.是，有一定程度 | B.有效 | A.非常清晰 | B. 是，有一定程度覆盖 | B.好 | A.很大提升 | A.是的，非常显著 | A.非常自信 | 它为我未来的职业角色提供了有效沟通和协作的关键技能。 | More collaborative learning tools could be integrated to help teams share insights more efficiently | Including topics related to post-trauma rehabilitation and physical therapy would provide a more complete view of patient care. |
| 59 | A.第一年 | C.儿童口腔医学 | B.满意 | B.是，有一定程度 | A.非常有效 | A.非常清晰 | B. 是，有一定程度覆盖 | B.好 | A.很大提升 | A.是的，非常显著 | B.自信 | 该课程的设计旨在增强理论理解和实践应用两方面的能力。 | The overall flow of sessions could be better structured to enhance engagement and reduce confusion. | More on forensic dentistry related to trauma cases would give students valuable insights into legal investigations. |
| 60 | A.第一年 | G.正畸科 | B.满意 | B.是，有一定程度 | B.有效 | A.非常清晰 | B. 是，有一定程度覆盖 | B.好 | A.很大提升 | A.是的，非常显著 | B.自信 | 我很喜欢这一模式促进了跨学科的互动，丰富了我的学习体验。 | More peer-reviewed articles could be integrated into the sessions to support evidence-based learning. | The inclusion of evidence-based practice and research in trauma management would help students stay updated with the latest knowledge. |
| 61 | B.第二年 | F.牙周病学 | A.非常满意 | B.是，有一定程度 | B.有效 | A.非常清晰 | B. 是，有一定程度覆盖 | B.好 | A.很大提升 | A.是的，非常显著 | B.自信 | 跨学科的重点让我更好地理解了创伤护理的医学、心理学和社会维度。 | Offering more opportunities for mentorship could strengthen student learning. | More focus on patient-centered care and ethics in trauma treatment would improve students' decision-making skills. |
| 62 | A.第一年 | E.修复科 | A.非常满意 | A.是的，非常显著 | B.有效 | A.非常清晰 | B. 是，有一定程度覆盖 | B.好 | A.很大提升 | A.是的，非常显著 | B.自信 | PBL课程帮助我建立了对牙外伤全面、整体的理解。 | More comprehensive training on ethics in trauma care would be valuable. | Offering more interdisciplinary team-building exercises would further enhance collaborative learning. |
| 63 | A.第一年 | D.牙体牙髓病科 | A.非常满意 | A.是的，非常显著 | B.有效 | A.非常清晰 | A.是，全面覆盖 | B.好 | A.很大提升 | A.是的，非常显著 | B.自信 | 在跨学科团队中工作帮助我培养了批判性思维，并适应不同专业视角。 | The program could better integrate technology in terms of digital trauma simulation tools. | The integration of trauma care within a public health context would help students understand broader impacts on communities. |
| 64 | A.第一年 | E.修复科 | A.非常满意 | A.是的，非常显著 | B.有效 | A.非常清晰 | A.是，全面覆盖 | B.好 | A.很大提升 | A.是的，非常显著 | B.自信 | 与来自不同领域的专业人士互动的机会帮助我拓展了知识，并优化了我对创伤护理的处理方法。 | Increasing the diversity of the case study scenarios would help improve preparedness for real-life challenges. | Incorporating topics related to dental trauma prevention programs would further enhance proactive care. |
| 65 | A.第一年 | G.正畸科 | A.非常满意 | A.是的，非常显著 | B.有效 | A.非常清晰 | A.是，全面覆盖 | B.好 | A.很大提升 | A.是的，非常显著 | B.自信 | PBL模式中培养的团队合作对发展沟通和解决问题的能力至关重要。 | There could be more practical exercises to reinforce the theoretical lessons. | Including advanced restorative techniques in dental trauma would expand students' clinical skill set. |
| 66 | A.第一年 | B.口腔颌面外科 | A.非常满意 | A.是的，非常显著 | B.有效 | A.非常清晰 | B. 是，有一定程度覆盖 | A.优秀 | B.是，有一定程度提升 | A.是的，非常显著 | A.非常自信 | 我在诊断方面取得了一些进步。通过学习和实践，我能够更准确地识别复杂创伤的体征和症状，提高了诊断的准确性和可靠性。（I have made some progress in diagnosis. Through study and practice, I have been able to more accurately identify the signs and symptoms of complex trauma, improving the accuracy and reliability of diagnosis.） | Balancing the need for autonomy in learning with providing sufficient guidance. | Enhance the assessment process by including interdisciplinary rubrics and peer evaluations |
| 67 | A.第一年 | B.口腔颌面外科 | A.非常满意 | A.是的，非常显著 | A.非常有效 | A.非常清晰 | A.是，全面覆盖 | B.好 | A.很大提升 | A.是的，非常显著 | B.自信 | 该项目的结构鼓励了批判性反思，并加深了对材料的投入。 | Clearer assessment criteria would help students understand how to improve in future sessions. | Adding sessions on interdisciplinary case management would help students learn how to navigate complex trauma scenarios. |
| 68 | B.第二年 | A.全科 | B.满意 | A.是的，非常显著 | A.非常有效 | B.清晰 | A.是，全面覆盖 | B.好 | B.是，有一定程度提升 | A.是的，非常显著 | B.自信 | PBL增强了我从多个角度分析复杂案例并协作寻找最佳解决方案的能力。 | A greater variety of interdisciplinary professionals should be included to provide broader insights. | Introducing interventional radiology and its role in trauma treatment could expand diagnostic capabilities. |
| 69 | A.第一年 | C.儿童口腔医学 | B.满意 | A.是的，非常显著 | A.非常有效 | B.清晰 | A.是，全面覆盖 | A.优秀 | B.是，有一定程度提升 | A.是的，非常显著 | B.自信 | 真实场景的设置帮助我建立了解决未来牙外伤所需的实践技能。 | It might be helpful to offer extra support for students who are struggling with interdisciplinary concepts. | More exposure to trauma care in military or conflict settings would prepare students for challenging environments. |
| 70 | B.第二年 | D.牙体牙髓病科 | B.满意 | A.是的，非常显著 | A.非常有效 | B.清晰 | A.是，全面覆盖 | A.优秀 | B.是，有一定程度提升 | A.是的，非常显著 | B.自信 | 跨学科的环境使我能够从全局视角发展新的病患治疗方法。 | A more hands-on approach to diagnostic techniques could help solidify the learning experience. | Including advanced forensic investigation techniques in trauma cases could provide practical legal knowledge. |
| 71 | C.第三年 | E.修复科 | B.满意 | A.是的，非常显著 | A.非常有效 | B.清晰 | A.是，全面覆盖 | A.优秀 | B.是，有一定程度提升 | A.是的，非常显著 | B.自信 | PBL提供了围绕牙外伤问题的全面视角，包括伦理方面的考量。 | The program could provide more information on the socio-economic impact of dental trauma. | Offering exposure to trauma care across different age groups, from pediatrics to geriatrics, could broaden learning. |
| 72 | A.第一年 | G.正畸科 | B.满意 | A.是的，非常显著 | A.非常有效 | B.清晰 | A.是，全面覆盖 | A.优秀 | B.是，有一定程度提升 | A.是的，非常显著 | B.自信 | PBL模式提供了一个独特的环境，我可以从同伴和导师那里学习。 | More interactive technology tools could help enhance group collaboration. | More hands-on experience with 3D modeling and simulation for dental trauma would improve diagnostic accuracy. |
| 73 | A.第一年 | C.儿童口腔医学 | B.满意 | A.是的，非常显著 | A.非常有效 | B.清晰 | A.是，全面覆盖 | A.优秀 | B.是，有一定程度提升 | A.是的，非常显著 | B.自信 | 我很感激有自由探索和按照自己的节奏学习的机会，同时仍然能得到专家的指导。 | A better balance between independent study and group work would help accommodate various learning styles. | More emphasis on mental health trauma and patient counseling would improve overall patient care. |
| 74 | C.第三年 | F.牙周病学 | B.满意 | A.是的，非常显著 | A.非常有效 | B.清晰 | A.是，全面覆盖 | A.优秀 | B.是，有一定程度提升 | A.是的，非常显著 | B.自信 | PBL为牙外伤的学习提供了现实背景，帮助我看到了理论概念的实际应用意义。 | Incorporating more fieldwork or exposure to real-world trauma settings could help make the learning more practical. | Including cross-disciplinary workshops on stress management and coping with trauma would benefit students. |
| 75 | C.第三年 | F.牙周病学 | B.满意 | A.是的，非常显著 | A.非常有效 | B.清晰 | A.是，全面覆盖 | A.优秀 | B.是，有一定程度提升 | A.是的，非常显著 | B.自信 | 小组中多样化的观点带来了创新的解决方案，并加深了对复杂案例的理解。 | A more structured schedule with checkpoints would help students track their progress more effectively. | The inclusion of trauma care in mobile or field settings would provide practical learning in challenging environments. |
| 76 | A.第一年 | C.儿童口腔医学 | B.满意 | A.是的，非常显著 | A.非常有效 | B.清晰 | A.是，全面覆盖 | A.优秀 | B.是，有一定程度提升 | A.是的，非常显著 | B.自信 | 以协作为基础的问题解决方法提高了我的学习能力和人际交往技能。 | Providing more visual aids and simulations would help with understanding complex dental trauma cases. | Offering sessions on trauma care in natural disasters would help students understand the complexities of large-scale trauma responses. |
| 77 | A.第一年 | E.修复科 | B.满意 | A.是的，非常显著 | A.非常有效 | B.清晰 | A.是，全面覆盖 | A.优秀 | B.是，有一定程度提升 | A.是的，非常显著 | B.自信 | 我发现跨学科合作对帮助我更好地理解创伤护理至关重要。 | Offering more interdisciplinary seminars could help increase the integration of knowledge across fields. | The introduction of virtual reality simulations for dental trauma treatment would make learning more interactive. |
| 78 | B.第二年 | D.牙体牙髓病科 | B.满意 | A.是的，非常显著 | A.非常有效 | B.清晰 | A.是，全面覆盖 | A.优秀 | B.是，有一定程度提升 | A.是的，非常显著 | B.自信 | 能够从多样化学生群体的讨论中学习并贡献自己的观点是非常宝贵的。 | Increased opportunities for practical skills workshops would be valuable for gaining hands-on experience. | Offering trauma care sessions in a global health context could enhance learning for students interested in international healthcare. |
| 79 | A.第一年 | B.口腔颌面外科 | B.满意 | A.是的，非常显著 | A.非常有效 | B.清晰 | A.是，全面覆盖 | A.优秀 | B.是，有一定程度提升 | A.是的，非常显著 | B.自信 | 在跨学科团队中工作帮助我理解了牙外伤的复杂性，以及不同领域如何为治疗作出贡献。 | More peer-to-peer learning opportunities could enhance student engagement. | Focusing on the social determinants of health and their impact on trauma recovery could offer a more holistic view of care. |
| 80 | A.第一年 | C.儿童口腔医学 | B.满意 | A.是的，非常显著 | A.非常有效 | B.清晰 | A.是，全面覆盖 | A.优秀 | B.是，有一定程度提升 | A.是的，非常显著 | B.自信 | PBL模式鼓励积极参与和互动，使学习更加生动有趣。 | The use of technology for remote case study discussions could help students stay engaged outside the classroom. | Including more on patient care continuity in trauma management could help students understand the long-term effects of trauma. |
| 81 | A.第一年 | F.牙周病学 | B.满意 | A.是的，非常显著 | A.非常有效 | B.清晰 | A.是，全面覆盖 | A.优秀 | B.是，有一定程度提升 | A.是的，非常显著 | B.自信 | PBL项目的跨学科性质帮助我培养了更全面的技能。 | More time for informal discussions between disciplines could improve collaborative understanding. | Offering interdisciplinary case studies with other healthcare professionals could improve the collaborative aspect of care. |
| 82 | A.第一年 | D.牙体牙髓病科 | B.满意 | A.是的，非常显著 | A.非常有效 | B.清晰 | A.是，全面覆盖 | A.优秀 | B.是，有一定程度提升 | A.是的，非常显著 | B.自信 | 学习如何在跨学科团队中工作帮助我发展了技术能力和软技能。 | It would be helpful to provide more clarification on the roles of each professional within the interdisciplinary teams. | A focus on oral and facial reconstructive surgery following trauma would be beneficial for a deeper understanding of dental trauma care. |
| 83 | A.第一年 | C.儿童口腔医学 | C.中立 | C.中立 | C.中立 | C.中立 | C.中立 | C.中立 | C.中立 | C.中立 | C.中立 | 在学习和借鉴他人解决问题的思路方面，我收获很多 | no | no |
| 84 | A.第一年 | E.修复科 | C.中立 | B.是，有一定程度 | C.中立 | C.中立 | C.中立 | C.中立 | C.中立 | C.中立 | C.中立 | 反复的思考和推敲有助于形成并完善我看待问题的逻辑 | no | no |
