# Supplementary material for: Interdisciplinary problem-based learning model for standardized dental residency training: from theory to practice in dental trauma management
Source: Front Med (Lausanne). 2025 Jan 13;11:1473943. doi: 10.3389/fmed.2024.1473943 (PMC11770602; doi:10.3389/fmed.2024.1473943)
Supplement: Supplementary file 6 [file Table_6.docx]

**Supplementary material 6. Course feedback from mentors**

|  | Q1 | Q2 | Q3 | Q4 | Q5 | Q6 | Q7 | Q8 | Q9 | Q10 | Q11 | Q12 |
| --- | --- | --- | --- | --- | --- | --- | --- | --- | --- | --- | --- | --- |
| 1 | 全科 | 5 | D.不同意 | B.有效 | E.否，没有 | E.非常不好 | E. 否，完全没有覆盖 | C.中立 | B.经常 | 不同学科之间协调时间表，确保所有成员参与。 | 制定统一的时间表以更好地协调不同学科之间的合作。 | D.不满意 |
| 2 | C.儿童口腔医学 | 4 | A.非常同意 | A.非常有效 | C.中立 | A.优秀 | A.是，全面覆盖 | A.非常有效 | A.总是 | 在复杂的跨学科案例中保持学生的参与和动力。 | 引入角色扮演练习，帮助学生理解不同专业的视角。 | B.满意 |
| 3 | D.牙体牙髓病科 | 6 | B.同意 | A.非常有效 | A.是的，非常显著 | A.优秀 | A.是，全面覆盖 | A.非常有效 | C.有时 | 学生对跨学科协作重要性的认识有限。 | 与学生的互动启发了新的想法和见解，应大力加强这方面的工作（Interaction with the residents inspired new thoughts and insights, highlighting the need for further enhancement in this area.） | A.非常满意 |
| 4 | E.修复科 | 3 | C.中立 | C.中立 | A.是的，非常显著 | C.中立 | C.中立 | D.无效 | D. 很少 | 难以将心理学或伦理学等非牙科领域整合到PBL案例中。 | 整合反思性练习，帮助学生理解协作的重要性。 | C.中立 |
| 5 | F.牙周病学 | 4 | C.中立 | C.中立 | C.中立 | C.中立 | D.否，不够多 | C.中立 | B.经常 | 应对诸如协作课程空间安排等后勤问题。 | 增加用于跨学科学习工具和资源的资金支持。 | C.中立 |
| 6 | D.牙体牙髓病科 | 5 | A.非常同意 | A.非常有效 | C.中立 | B.好 | A.是，全面覆盖 | B.有效 | C.有时 | 管理小组每位同学的基本情况及动态，确保每位学生都有公平的机会参与 | 为教师提供培训课程，提高他们在跨学科环境中的引导能力。 | B.满意 |
| 7 | E.修复科 | 2 | C.中立 | C.中立 | B.是，有一定程度 | B.好 | D.否，不够多 | B.有效 | B.经常 | 在内容深度和跨学科广度之间取得平衡，以实现有效的协作。 | 引入更多实践性、模拟化的案例以更好地结合理论与实践。 | A.非常满意 |
| 8 | A.全科 | 3 | D.不同意 | E.非常无效 | B.是，有一定程度 | B.好 | D.否，不够多 | B.有效 | B.经常 | 确保学生能够学习并应用其他学科的知识。 | 使用医院或诊所提供的真实案例研究以增强真实性 | D.不满意 |
| 9 | A.全科 | 5 | B.同意 | B.有效 | A.是的，非常显著 | C.中立 | C.中立 | B.有效 | C.有时 | 缺少能够提升跨学科学习的软件或工具。 | 提供可选的跨学科选修课程以供深入学习。 | B.满意 |
| 10 | D.牙体牙髓病科 | 4 | D.不同意 | B.有效 | D.否，不太多 | C.中立 | B. 是，有一定程度覆盖 | E.非常无效 | B.经常 | 确保不同小组之间跨学科PBL的实施一致性。 | 使用标准化患者来进行跨学科情境下的真实案例模拟。 | A.非常满意 |
| 11 | D.牙体牙髓病科 | 6 | C.中立 | B.有效 | A.是的，非常显著 | B.好 | B. 是，有一定程度覆盖 | A.非常有效 | C.有时 | 难以找到涵盖多个学科的相关且真实的案例研究。 | 使用数字工具（如协作平台）改善沟通和资源共享。 | A.非常满意 |
| 12 | C.儿童口腔医学 | 8 | C.中立 | B.有效 | A.是的，非常显著 | A.优秀 | A.是，全面覆盖 | A.非常有效 | A.总是 | 建立不同学科学生之间的信任和有效沟通存在困难。 | 儿童牙外伤的发生在性别、年龄、季节等方面具有一定的规律性，儿童牙外伤往往不能及时就诊。加强儿童牙外伤的健康教育，对于降低牙外伤的发生概率至关重要（The occurrence of dental trauma in children has regularity in terms of gender, age, and seasons, and children often do not seek timely medical consultation for dental trauma. Strengthening health education on dental trauma in children is essential to reduce the probability of dental injuries.） | B.满意 |
| 13 | B.口腔颌面外科 | 7 | B.同意 | A.非常有效 | A.是的，非常显著 | D.不太好 | A.是，全面覆盖 | A.非常有效 | B.经常 | 如何恰当的反映跨学科学习的特性 | 通过校友反馈和案例复盘来评估PBL模式的长期成果。 | D.不满意 |
| 14 | C.儿童口腔医学 | 6 | B.同意 | D.无效 | D.否，不太多 | B.好 | B. 是，有一定程度覆盖 | A.非常有效 | C.有时 | 缺乏多领域专家参与案例讨论的资源。 | 邀请相关领域的专业人士进行客座讲座，提供实践性见解。 | A.非常满意 |
| 15 | C.儿童口腔医学 | 2 | B.同意 | C.中立 | B.是，有一定程度 | A.优秀 | C.中立 | D.无效 | A.总是 | 克服跨学科协作的障碍，例如学科间壁垒和不同的教学方法。 | 通过宏观计划和细节方面的调整，克服跨学科协作的障碍 | C.中立 |
| 16 | D.牙体牙髓病科 | 5 | B.同意 | B.有效 | A.是的，非常显著 | D.不太好 | B. 是，有一定程度覆盖 | A.非常有效 | B.经常 | 克服学生和教师对PBL结果的期望不一致之间的挑战。 | 提供案例后续跟进，展示PBL课程中决策的长期影响。 | B.满意 |
| 17 | G.正畸科 | 6 | B.同意 | B.有效 | A.是的，非常显著 | A.优秀 | A.是，全面覆盖 | A.非常有效 | C.有时 | 外伤牙早期固定正畸治疗的最佳方法和时机仍存在争议，应有更多的证据确定牙齿移动的影响、正畸加力的方向以及正畸应用的类型。（The optimal method and timing of early fixed orthodontic treatment for traumatized teeth are still controversial. Further evidence is needed to determine the effects of tooth movement, the direction of orthodontic force application, and the type of orthodontic appliance used. |  | A.非常满意 |
| 18 | E.修复科 | 3 | A.非常同意 | B.有效 | A.是的，非常显著 | B.好 | B. 是，有一定程度覆盖 | B.有效 | A.总是 | 缺乏能够有效连接多个学科的清晰学习目标或者平台 | 创建跨学科PBL专责小组，以协作开发和改进内容。 | A.非常满意 |
| 19 | F.牙周病学 | 4 | A.非常同意 | D.无效 | B.是，有一定程度 | C.中立 | D.否，不够多 | A.非常有效 | B.经常 | 确保所有学生理解其他学科在创伤护理中的角色。 | 查找并在调研基础上设计更多反映现实中跨学科挑战的多样化案例。 | B.满意 |
| 20 | G.正畸科 | 6 | B.同意 | A.非常有效 | B.是，有一定程度 | B.好 | C.中立 | A.非常有效 | B.经常 | 确保那些可能缺乏自信或不适应该模式的学生积极参与。 | 促进不同部门之间的更好协作，以创建更统一的课程。 | B.满意 |
| 21 | A.全科 | 8 | A.非常同意 | B.有效 | A.是的，非常显著 | B.好 | B. 是，有一定程度覆盖 | E.非常无效 | C.有时 | 教师缺乏足够的培训来有效引导跨学科PBL课程。 | 实施结构化的指导方针，明确每个学科在PBL案例中的角色。 | A.非常满意 |
| 22 | D.牙体牙髓病科 | 7 | A.非常同意 | B.有效 | D.否，不太多 | C.中立 | B. 是，有一定程度覆盖 | B.有效 | A.总是 | 难以公平评估学生在多个学科中的表现。 | 使用学生反馈不断优化PBL课程内容和交付方式。 | B.满意 |
| 23 | D.牙体牙髓病科 | 6 | A.非常同意 | A.非常有效 | A.是的，非常显著 | B.好 | A.是，全面覆盖 | A.非常有效 | B.经常 | 克服偏好传统课堂教学模式的学生的抵触情绪。 | 引入更多跨学科的工作坊和研讨会以加强协作。 | D.不满意 |
| 24 | D.牙体牙髓病科 | 3 | A.非常同意 | B.有效 | B.是，有一定程度 | D.不太好 | C.中立 | B.有效 | C.有时 | 时间有限，难以同时覆盖理论基础和跨学科协作内容。 | 制定清晰且可衡量的学习目标，涵盖个人和团队的成果。 | B.满意 |
| 25 | E.修复科 | 3 | B.同意 | A.非常有效 | A.是的，非常显著 | A.优秀 | B. 是，有一定程度覆盖 | B.有效 | B.经常 | 在PBL框架中整合新兴技术的困难和实施落地 | 分配更多时间用于跨学科讨论和案例总结。 | C.中立 |
| 26 | F.牙周病学 | 4 | A.非常同意 | A.非常有效 | B.是，有一定程度 | A.优秀 | C.中立 | A.非常有效 | C.有时 | 难以为所有学生提供充足的资源以深入探索跨学科主题。 | 为较不熟悉的学科提供额外资源，例如阅读材料或视频教程。 | B.满意 |
| 27 | G.正畸科 | 6 | A.非常同意 | B.有效 | B.是，有一定程度 | C.中立 | A.是，全面覆盖 | A.非常有效 | C.有时 | 应对来自不同学科的学生在知识和技能水平上的差异。 | 提供激励措施（如证书），以激励学生和教师充分参与。 | B.满意 |
| 28 | E.修复科 | 5 | A.非常同意 | B.有效 | B.是，有一定程度 | A.优秀 | B. 是，有一定程度覆盖 | B.有效 | A.总是 | 缺乏支持开发高质量跨学科案例材料的资金。 | 创建更小、更专注的小组以提高讨论和参与的质量。 | B.满意 |
| 29 | F.牙周病学 | 5 | B.同意 | A.非常有效 | A.是的，非常显著 | C.中立 | A.是，全面覆盖 | A.非常有效 | C.有时 | 在提供足够指导和保持学习自主性之间取得平衡。 | 通过增加跨学科评分标准和同伴评价来增强评估流程。 | A.非常满意 |
| 30 | G.正畸科 | 2 | B.同意 | B.有效 | A.是的，非常显著 | B.好 | B. 是，有一定程度覆盖 | A.非常有效 | C.有时 | 客观评估跨学科PBL模式效果的难度。 | 引入虚拟现实等新兴技术以模拟复杂的创伤案例。 | A.非常满意 |
| 31 | A.全科 | 7 | A.非常同意 | A.非常有效 | A.是的，非常显著 | B.好 | B. 是，有一定程度覆盖 | B.有效 | B.经常 | 准备和协调跨学科PBL课程时面临时间限制。 | 在有条件的基础上建立导师计划，让学生可以向来自不同领域的专业人士学习。 | A.非常满意 |
| 32 | D.牙体牙髓病科 | 8 | A.非常同意 | A.非常有效 | A.是的，非常显著 | B.好 | A.是，全面覆盖 | B.有效 | C.有时 | 不同部门的教师之间缺乏充分的协作。 | 在跨学科团队中培养开放沟通和相互尊重的文化。 | D.不满意 |
