# Supplementary material for: Interdisciplinary problem-based learning model for standardized dental residency training: from theory to practice in dental trauma management
Source: Front Med (Lausanne). 2025 Jan 13;11:1473943. doi: 10.3389/fmed.2024.1473943 (PMC11770602; doi:10.3389/fmed.2024.1473943)
Supplement: Supplementary file 7 [file Table_7.docx]

**Supplementary material 7. Bootstrap for Independent Samples Test**

|  | Scores | t | | p | 95% Confidence Interval | |
| --- | --- | --- | --- | --- | --- | --- |
|  |  |  |  |  | Lower | Upper |
| Experimental groups | 89.82±8.08 | | -5.124 | 0.001 | -7.42625 | -3.40542 |
| Control groups | 84.42±9.14 | | |  |  |  |
